# EVApeCognition: An 18-Year Dataset of Great Ape Cognition

**DOI:** 10.1038/s41597-026-07191-6

**Published:** 2026-04-09

**Authors:** Alejandro Sánchez-Amaro, Sonja J. Ebel van Wijk, Carin Molenaar, Akzira Abuova, Lizbeth Mujica-Manrique, Sarah M. Leisterer-Peoples, Bret Beheim, Luke Maurits, Anna Albiach-Serrano, Matthias Allritz, Nazli Altınok, Federica Amici, Alice MI Auersperg, Filippo Aureli, Elisa Bandini, Jochen Barth, Leïla Benziad, Bettina E. Bläsing, Manuel Bohn, Marie Bourjade, Juliane Bräuer, Marie-Hélène Broihanne, Sarah F. Brosnan, Nereida Bueno-Guerra, Thomas Bugnyar, David Buttelmann, Frances Buttelmann, Trix Cacchione, Malinda Carpenter, Fernando Colmenares, Catherine Crockford, Katherine A. Cronin, África de las Heras, Arianna De Marco, Sarah E. DeTroy, Valérie Dufour, Shona Duguid, Robin I. M. Dunbar, Johanna Eckert, Jan M. Engelmann, Joel Fagot, Julia Fischer, Sofia Ingrid Fredrika Forss, Martina Funk, György Gergely, Julia R. Greenberg, Johannes Großmann, Sebastian Grüneisen, Marta Halina, Daniel Hanus, Sarah R. Heilbronner, Christophe Heintz, Robert Hepach, Esther Herrmann, Satoshi Hirata, Alenka Hribar, Gabriele Janzen, Juliane Kaminski, Patricia Kanngiesser, Fumihiro Kano, Katharina C. Kirchhofer, Hagen Knofe, Kathrin S. Kopp, Christopher Krupenye, Isabelle Barbara Laumer, Stephen C. Levinson, Ulf Liszkowski, Héctor M. Manrique, Gema Martin-Ordas, Emma Suvi McEwen, Richard T. Moore, Enric Munar, Marcos Nadal, Christian Nawroth, Suska Nolte, Marie Pelé, Patrizia Potì, Hannes Rakoczy, Julia Riedel, Amélie Romain, Federico Rossano, Yvan I. Russell, Gloria Sabbatini, Marie Schäfer, Marina Scheumann, Martin Schmelz, Benjamin Schmid, Vanesa Schmitt, Carla Sebastián-Enesco, Amanda Madeleine Seed, Chikako Suda-King, Tibor Tauzin, Sebastian Tempelmann, Claudio Tennie, Valentina Truppa, Jana Uher, Amrisha Vaish, Edwin J.C. van Leeuwen, Elisabetta M. Visalberghi, Christoph J. Völter, Victoria Vonau, Claudia A. F. Wascher, Roman M. Wittig, Wouter Wolf, Michael Tomasello, Katja Liebal, Josep Call, Daniel B. M. Haun

**Affiliations:** 1 Division of Psychology, Faculty of Natural Sciences, University of Stirling, Stirling, UK; 2 Department of Comparative Cultural Psychology, Max Planck Institute for Evolutionary Anthropology, Leipzig, Germany; 3 Human Biology and Primate Cognition, Institute of Biology, Faculty of Life Sciences, Leipzig University, Leipzig, Germany; 4 Department of Human Behavior, Ecology and Culture, Max Planck Institute for Evolutionary Anthropology, Leipzig, Germany; 5 Ethology and Animal Welfare Section, Universidad Cardenal Herrera-CEU, CEU Universities, Valencia, Spain; 6 Department of Psychology, University of Konstanz, Konstanz, Germany; 7 Department of Interdisciplinary Life Sciences/Messerli Research Institute/Comparative Cognition Unit, University of Veterinary Medicine, Vienna, Austria; 8 Instituto de Neuroetología, Universidad Veracruzana, Xalapa, Mexico; 9 Research Centre in Evolutionary Anthropology and Palaeoecology, Liverpool John Moores University, Liverpool, UK; 10 Department of Evolutionary Biology and Environmental Studies, The University of Zürich, Zürich, Switzerland; 11 Interdisciplinary Center for Archaeology and the Evolution of Human Behaviour, Universidade do Algarve, Faro, Portugal; 12 Department Networks and Transfer, Fraunhofer Institute for Machine Tools and Forming Technology, Dresden, Germany; 13 Department of Developmental and Comparative Psychology, Max Planck Institute for Evolutionary Anthropology, Leipzig, Germany; 14 Department of Sport Science, Bielefeld University, Bielefeld, Germany; 15 Institute for Psychology in Education, Leuphana University Lüneburg, Lüneburg, Germany; 16 Laboratoire CLLE, UMR CNRS 5263, Université de Toulouse Jean Jaurès, Institut Universitaire de France, Toulouse, France; 17 Department for General Psychology and Cognitive Neuroscience, Institute of Psychology, Friedrich Schiller University of Jena, Jena, Germany; 18 DogStudies, Max Planck Institute of Geoanthropology, Jena, Germany; 19 LaRGE EM Strasbourg, University of Strasbourg, Strasbourg, France; 20 Department of Psychology, Neuroscience Institute, and Language Research Center, Georgia State University, Atlanta, GA USA; 21 Departamento de Psicología, Universidad Pontificia Comillas, Madrid, Spain; 22 Department of Behavioral and Cognitive Psychology, University of Vienna, Vienna, Austria; 23 Konrad Lorenz Research Station, Core Facility, University of Vienna, Grünau, Austria; 24 Department of Developmental Psychology, Institute of Psychology, University of Bern, Bern, Switzerland; 25 Center for Lifespan Developmental Science (CELISE), Friedrich Schiller University of Jena, Jena, Germany; 26 Developmental Psychology, Institute of Primary Education, University of Teacher Education FHNW, Brugg-Windisch, Switzerland; 27 School of Psychology and Neuroscience, University of St Andrews, St Andrews, UK; 28 Departamento de Psicobiología y Metodología en Ciencias del Comportamiento, Facultad de Psicología, Campus de Somosaguas, Universidad Complutense de Madrid, Madrid, Spain; 29 The Ape Social Mind Lab, Institute for Cognitive Sciences Marc Jeannerod, CNRS, Lyon, France; 30 Animal Welfare Science Program, Lincoln Park Zoo, Chicago, IL USA; 31 Fondazione Ethoikos, Radicondoli, Italy; 32 Parco Faunistico di Piano dell’Abatino, Poggio San Lorenzo, Italy; 33 Laboratoire de Psychology Sociale et Cognitive, University of Clermont Auvergne & Centre National pour la Recherche Scientifique, Clermont-Ferrand, France; 34 School of Education, Language and Psychology, York St John University, York, UK; 35 Department of Experimental Psychology, University of Oxford, Oxford, UK; 36 Department for the Ecology of Animal Societies, Max Planck Institute of Animal Behavior, Konstanz, Germany; 37 Department of Psychology, University of California Berkeley, Berkeley, CA USA; 38 Centre de Recherche en Psychologie et Neurosciences (CRPN), CNRS, Aix-Marseille Université, Marseille, France; 39 Station de Primatologie-Celphedia, CNRS UAR846, Rousset, France; 40 Cognitive Ethology Lab, German Primate Center - Leibniz Institute for Primate Research, Goettingen, Germany; 41 Department of Primate Cognition, Georg-August-University Göttingen, Göttingen, Germany; 42 Zoologisches Institut der Universität Zürich, Abteilung Ethologie und Wildforschung, Zürich, Switzerland; 43 Cognitive Development Center, Department of Cognitive Science, Central European University, Vienna, Austria; 44 Department of Psychology, University of Wisconsin- Madison, Madison, WI USA; 45 Faculty of Education, Leipzig University, Leipzig, Germany; 46 Department of History and Philosophy of Science, University of Cambridge, Cambridge, UK; 47 Department of Neurosurgery, Baylor College of Medicine, Houston, TX USA; 48 Centre for Comparative and Evolutionary Psychology, School of Psychology, Sport and Health Sciences, University of Portsmouth, Portsmouth, UK; 49 Wildlife Research Center, Kyoto University, Kyoto, Japan; 50 Behavioural Science Institute, Radboud University, Nijmegen, The Netherlands; 51 Donders Institute for Brain, Cognition and Behaviour, Radboud University, Nijmegen, The Netherlands; 52 School of Psychology, University of Plymouth, Plymouth, UK; 53 Center for the Advanced Study of Collective Behavior, University of Konstanz, Konstanz, Germany; 54 Department of Psychological & Brain Sciences, Johns Hopkins University, Baltimore, MD USA; 55 Development and Evolution of Cognition Research Group, Max Planck Institute of Animal Behavior, Konstanz, Germany; 56 Language Development Department, Max Planck Institute for Psycholinguistics, Nijmegen, The Netherlands; 57 Developmental Psychology, University of Hamburg, Hamburg, Germany; 58 Departamento de Psicología y Sociología, Universidad de Zaragoza, Teruel, Spain; 59 Department of Psychology, University of Girona, Girona, Spain; 60 Department of Philosophy, University of Warwick, Coventry, UK; 61 Human Evolution and Cognition Research Group (EvoCog), University of the Balearic Islands, Palma de Mallorca, Spain; 62 Working group “Animal Behaviour & Welfare”, Research Institute for Farm Animal Biology, Dummerstorf, Germany; 63 Anthropo-Lab ETHICS EA 7446, Université Catholique de Lille, Lille, France; 64 Institute of Cognitive Sciences and Technologies, National Research Council of Italy, Rome, Italy; 65 Department of Developmental Psychology, Institute of Psychology, University of Göttingen, Göttingen, Germany; 66 Wild Chimpanzee Foundation, Abidjan, Ivory Coast; 67 Bureau d’études AKONGO, Nantes, France; 68 Department of Cognitive Science, University of California San Diego, San Diego, CA USA; 69 Department of Psychology, Middlesex University, London, UK; 70 Wilhelm Wundt Institute for Psychology, Leipzig University, Leipzig, Germany; 71 Institute of Zoology, University of Veterinary Medicine Hannover, Hannover, Germany; 72 Department of Child and Adolescent Psychiatry and Psychotherapy, University Medical Center Göttingen, Göttingen, Germany; 73 UCM Research Group of Social, Developmental and Comparative Psychobiology, Faculty of Psychology, Universidad Complutense de Madrid, Madrid, Spain; 74 Lexical Intelligence, Rockville, MD USA; 75 Department of Linguistics, University of Vienna, Vienna, Austria; 76 Institute for Research and Development, University of Teacher Education Bern, Bern, Switzerland; 77 Faculty of Science, Department of Geosciences, Working Group Early Prehistory and Quaternary Ecology, University of Tübingen, Tübingen, Germany; 78 School of Human Sciences, University of Greenwich, London, UK; 79 Department of Psychology, University of Virginia, Charlottesville, VA USA; 80 Animal Behaviour and Cognition, Department of Biology, Utrecht University, Utrecht, The Netherlands; 81 Behavioural Ecology Research Group, School of Life Sciences; Anglia Ruskin University, Cambridge, UK; 82 Department of Primatology, Max Planck Institute for Evolutionary Anthropology, Leipzig, Germany; 83 Department of Developmental Psychology, Utrecht University, Utrecht, The Netherlands; 84 Department of Psychology and Neuroscience, Duke University, Durham, NC USA

## Abstract

The study of great ape cognition offers insights into the evolutionary origins of human intelligence, but is hindered by small sample sizes and restricted access to data. To address this, we present the EVApeCognition Dataset, a publicly available resource comprising 262 experimental datasets from 150 scientific publications from the Wolfgang Köhler Primate Research Center (2004–2021) in Leipzig, Germany. Eighty-one apes participated in 150 studies, with a majority (N = 78) participating in more than one study. Publication of the dataset aims to make these unique datasets accessible for future meta-analyses and correlational analyses, helping us better understand how our great ape relatives think, learn, and behave.

## Introduction

As members of the Hominidae family, humans share a recent evolutionary history with other great apes - chimpanzees, bonobos, gorillas, and orangutans. Among these, chimpanzees and bonobos are our closest living relatives, having diverged from a common ancestor with humans only around six million years ago^1^. This close phylogenetic relationship makes the study of great apes a powerful lens through which to explore our own evolutionary roots: By identifying which traits are shared with other apes due to common ancestry and which are species-specific, we gain important insights into the characteristics that make humans human.

Behavioral observations of great apes have long provided valuable insights into their social lives, problem-solving abilities, and ecological strategies^2–5^. Studying cognition — the mental processes underlying observable behavior — enables researchers to construct powerful explanatory models of how (in Tinbergen’s proximate sense) species behave as they do in addition to describing what they do^6^. This cognitive lens is especially critical in comparative research that seeks to uncover the evolutionary origins of complex psychological capacities—such as abstract reasoning, perspective-taking, and metacognition—that characterize our species.

Over the past few decades, research into the cognition of chimpanzees, bonobos, gorillas, and orangutans has significantly advanced our understanding of how our closest living relatives behave, think, and learn^7–13^. Studies on physical (self in the world) and social cognition (self and others) have provided valuable insights into whether cognitive capacities like, for example, theory of mind (the ability to represent others’ mental states)^11^, metacognition (monitoring and representing one’s own mental states)^14,15^, prosocial concern (the motivation to help others)^16^, and the understanding of cause-and-effect relationships (understanding how certain actions in the world produce specific outcomes)^17^, are shared with our closest living relatives or are species-specific. This body of research has also shed light on how domain-general executive functions such as working memory, inhibition, and cognitive flexibility regulate great apes’ decisions in cognitive tasks^18,19^. Studies like these have laid the groundwork for many influential theories of human evolution^20–25^, relational thought^26^, and causal reasoning^27^, reshaping our understanding of the emergence of uniquely human cognition.

Despite this progress, cognitive research in great apes still faces practical limitations. The majority of studies conducted in zoos and other captive environments often involves small populations. Participation in cognitive tasks is strictly voluntary, resulting in even more selective and reduced sample sizes. The limited number of data points restricts our ability to investigate variation across individuals, age groups, and socio-ecological contexts, ultimately constraining generalizability and replicability^28–30^.

Large-scale collaborative efforts have emerged to address these challenges^31^. In particular, the *ManyPrimates* initiative has brought together researchers interested in primate cognition to gain a richer and more robust perspective on the cognitive abilities of our closest living relatives^32,33^–see also^34,35^ for similar initiatives with other animal groups. However, along with sampling widely across species, we also need detailed, long-term in-depth investigations into the ontogeny, individual differences, and the structure of cognition across tasks within a species—dimensions that are difficult, if not impossible, to capture in single studies.

By aggregating 262 experimental datasets from 150 publications^16,32,36–183^, the *EVApeCognition Dataset*, presented here, enables the study of cognitive structure, its ontogeny and variation in great apes at an unprecedented scale. Furthermore, the dataset will continue growing after its publication (see the list of studies in the Supplementary Text (ST)) to preserve invaluable data for current and future generations. All studies were conducted at one of the leading and most prolific^184,185^ institutions studying captive great ape cognition and behavior: the Wolfgang Köhler Primate Research Center (WKPRC). The WKPRC was established in 2001 by Leipzig Zoo and Max Planck Institute for Evolutionary Anthropology in Leipzig, Germany (MPI-EVA). Based on studies conducted at the WKPRC, we created the EVApeCognition Dataset - the currently largest and most comprehensive collection of experimental studies of great apes’ cognition and behavior. The dataset complies with the FAIR (Findable, Accessible, Interoperable, Reusable) data management principles and sharing practices^186^. Every data file in this searchable and open-access dataset has been validated by primary sources, formatted following the same structure, and it is published along with metadata information and a glossary of terminology to facilitate its usage.

The EVApeCognition Dataset represents data on a wide range of cognitive domains^7,8^, enabling researchers to investigate long-term trends and tackle longitudinal questions that single studies struggle to address, such as developmental milestones^187^, relations across cognitive tasks and domains^188,189^, and building computational models^190^. We hope this dataset will inspire other institutions to adopt similar practices to store and curate open access datasets that allow researchers collectively build a better understanding of great apes cognition and behavior.

## Materials and Methods

### Procedure

**Fig. 1:**
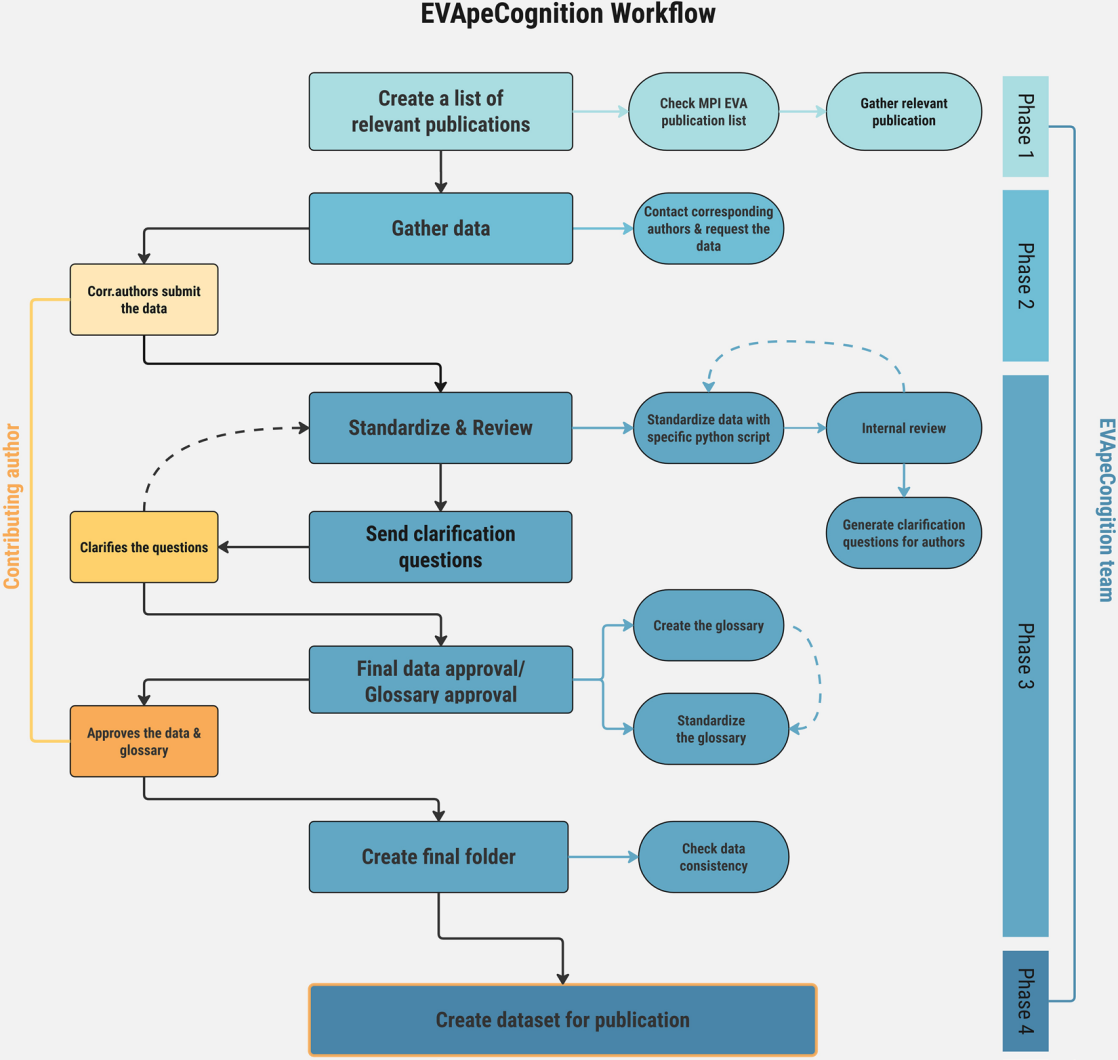
The four main phases in creating the EVApeCognition Dataset and the general workflow in each step.

The EVApeCognition Dataset was created in four main phases (see Fig. 1). In phase 1, we collated a list of the relevant publications. In phase 2, we gathered the data. In phase 3, we standardized and conducted a rigorous internal review of the data. Finally, in phase 4, we compiled all publishable datasets and created the dataset. Each of these phases is described in more detail in the subsequent sections.

#### Phase 1

All published experimental studies conducted with great ape participants at the WKPRC were considered eligible for inclusion in the dataset. An initial list of relevant publications, compiled in 2021 by S.EvW. and A.S., was generated using a set of keywords related to primates (e.g., *primate*, *ape*, *affe [German for “ape”]*, *chimpanzee*, *chimp*, *bonobo*, *gorilla*, *orang*, *orangutan*, *pan*, *troglodytes*, *paniscus*, *pongo*, *abelii*, *nonhuman*, *non-human*, *hominid*) and comparative research (e.g., *comparative*, *cognition*, *species*). The compilation process was supported by the library at the MPI-EVA. The resulting list of studies was subsequently filtered based on the following inclusion criteria:

1. Exclusion of purely observational studies with no experimental data to facilitate comparability between studies
2. Exclusion of unpublished studies and datasets. Unpublished datasets related to published studies were included at the discretion of the corresponding authors. The main reason for excluding unpublished studies was the difficulty of validating and standardizing those datasets with the same degree of precision. Many challenges arise from standardizing non-published data, including difficulties in identifying the identity of corresponding authors (if datasets were readily available at the MPI-EVA servers), validation of study participants, procedures, and identification of original datasets.
3. Exclusion of human child or adult subjects’ data from any dataset.
4. Exclusion of studies with only non-WKPRC participants. We excluded studies only containing non-WKPRC participants because our focus was to build a dataset of studies conducted at the WKPRC. Furthermore, the available information about non-WKPRC participants lacked the level of detail for consistent and reliable analysis compared to WKPRC participants.
5. Partial exclusion of non-WKPRC participants from studies containing WKRPC participants (non-WKPRC participants data was included at the discretion of the corresponding authors, and datasets with excluded participants were labelled accordingly).

The initial list of publications contained 246 published studies published between 2004 and 2021.

#### Phase 2

Once the initial list of publications was compiled, corresponding authors were contacted wherever possible (i.e. an informative email was successfully delivered to the authors’ email address), covering 97.5% of identified published studies. Authors were informed of the aims of the project, invited to online informational meetings (September-November 2021), and asked if they were interested in contributing the data of their published studies conducted at the WKPRC. To acknowledge contributions, we offered co-authorship to any corresponding author who provided their data.

In many cases, authors’ datasets were already archived in the MPI-EVA servers. In those cases, we contacted the corresponding authors to seek clarification and obtain permission to use the archived data (see “Technical Validation – Original Data” for further details). In addition to test data, authors were welcome to contribute supplementary datasets such as training or pretest data, or data from participants at other institutions (e.g., zoos or sanctuaries), at their discretion. Corresponding authors were responsible for informing their co-authors about the project. However, if a corresponding author could not be reached, co-author (s) were also invited to contribute. Authors were given a generous submission window, from November 1, 2021, through December 31, 2024, to facilitate contributions. In some cases, co-authors assumed the role of leading authors for purposes of standardization and review when corresponding authors were unavailable. Nevertheless, for consistency, we will use the term corresponding authors inclusively to refer to all authors actively involved in building the dataset.

#### Phase 3

After the corresponding authors provided the raw original data for each publication, the datasets were standardized according to criteria developed by A.S., and S.EvW., with the assistance of J.C., D.H., and K.L. (see “Technical Validation - Standardization” for more information). Following the initial standardization, all datasets underwent a rigorous internal review (see “Technical Validation – Internal Review” for more information). Multiple rounds of standardization and review were conducted as needed, based on the judgment of the internal reviewers. Throughout this process, corresponding authors were consulted to ensure data accuracy and consistency. After the data were fully standardized and the internal review process was complete, corresponding authors were asked to review, provide feedback, and approve the final standardized data and corresponding descriptive information for the glossaries associated with their data. For datasets included at the time of publication, corresponding authors were given until the 31^st^ of March 2025 to complete their review. Once all the standardized datasets were reviewed and approved, they were incorporated into the dataset. Each study entry includes a final dataset and glossary per experiment unless otherwise specified. Additionally, each study is also accompanied by a YAML file containing the relevant public metadata. See the ST for an example of a YAML file with the full record of time-stamped events and correspondence during the standardization process, and a publication-ready version containing only the metadata intended for public release.

#### Phase 4

**Fig. 2:**
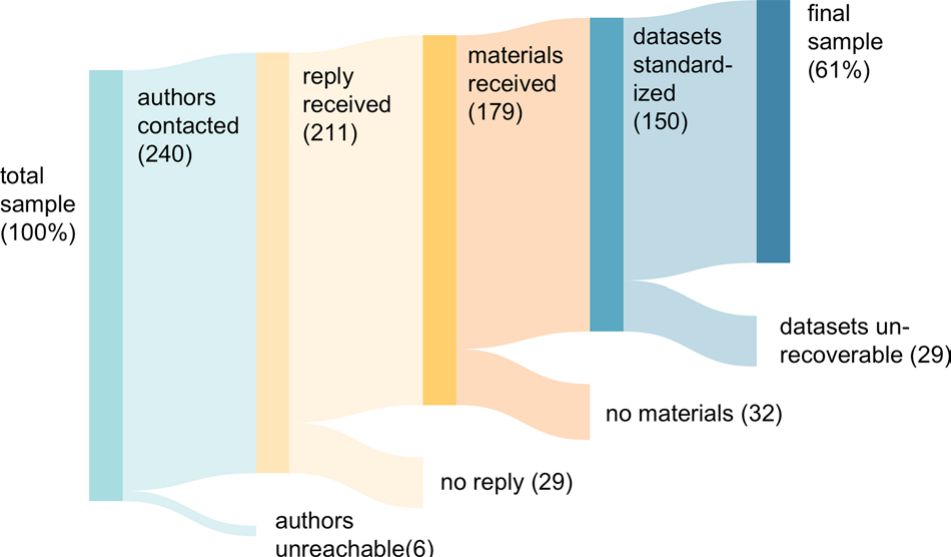
Sankey plot visualizing the step-by-step process of data recovery from total to final sample.

At the time of publication, the final dataset included data for 150 of 246 published studies–61% of the studies included in the final publication list (See Fig. 2). Even though the dataset lacks several studies, the percentage of recovered studies is very high when compared to previous initiatives in human psychology^191,192^. Nonetheless, data availability decreased for older publications, with more recent studies being more likely to have recoverable datasets, which aligns with what was seen by Minocher and colleagues^191^ in a related project in evolutionary anthropology. A complete overview of the included studies and the percentage of recovered studies over time can be found in the ST. Upon the completion of all final data folders, we created the EVApeCognition Dataset repository (https://github.com/ccp-eva/EVApeCognition). Importantly, this dataset publication is just the first step. The EVApeCognition Dataset has been conceived as a continuous project, allowing researchers to contribute datasets from studies conducted at the WKPRC, so this invaluable resource continues to grow alongside the literature. The next step would be to include data from studies published after 2021. We also envision the possibility of adding observational data in future iterations to combine it with the experimental data present in this dataset.

### Inclusion and ethics statement

All data used in this project are either publicly available or were provided as part of previously published research. Each original dataset standardized for inclusion in the EVApeCognition Dataset received ethical approval from the relevant institutional review boards or ethics committees at the time of data collection. Additional unpublished datasets, included in the EVApeCognition Dataset with permission of the corresponding authors (see Phase 2), were collected under the ethical approval of their home institutions. No new data was collected specifically for this publication.

## Data Records

The dataset is curated in a public GitHub repository (https://github.com/ccp-eva/EVApeCognition) and published in Zenodo^193^. The status of the repository at any given time can be browsed using tag-specific URLs (e.g., https://github.com/ccp-eva/EVApeCognition/releases/tag/v1.0.4). Changes between releases can always be compared https://github.com/ccp-eva/EVApeCognition/tags.

Released versions of the dataset are accessible and archived in 10.5281/zenodo.18846348 (this DOI will always direct to the latest version of the dataset). The choice of this platform provides us with permanent DOI for citing specific versions of the dataset. Zenodo provides metadata to identify earlier released versions.

The dataset is organized by publication, with each publication folder uniquely identified by a combination of the last name of the first author, the year of publication, and the first word in the publication title.

Each folder within study_files contains the following files:

- Metadata file (.yaml)
  - Full reference: Complete bibliographic citation of the publication.
  - Author list: All listed authors from the original publication.
  - Published abstract: As it appears in the original article.
  - Domain classifications: Cognitive domain (social or physical), general domain, and specific domain—curated by A.S. and J.C. based on current standards in the field^7,8^.
  - Experiment-level metadata: Metadata entries for each experiment included in the publication.
- Standardized data file(s) (.csv)
  - One or more standardized datasets, organized per experiment.
- Glossary file(s) (.csv)
  - A corresponding glossary for each dataset, describing the variables included in the standardized data files (see Table 1).

**Table 1:** Glossary template with definitions for all standardized variables.

column_name	Description
study_id	personal study ID composed by the first authors’ first surname_year of publication_first title word
Experiment	number of experiment from publication
experiment_name	name of data subset – can include different experiments within data set/ different training conditions/ pre-test data/ etc
Year	year the data point was obtained
Month	month the data point was obtained
Day	day of the month the data point was obtained
Participant	name of the participant
age_original	age provided by author in data file
age_in_years	age presented in years – see metadata for source of age
Sex	sex of the participant
Role	role of the participant – focal_participant/partner/stooge/etc/
participant_2*	name of the participant
age_in_years_2*	age presented in years – see metadata for source of age
sex_2*	sex of the participant
role_2*	role of the participant – focal_participant/partner/stooge/etc/
Present	another individual present but not participating – can be an infant or in rare cases an adult
Species	species of individuals participating in the study -bonobo/chimpanzee/gorilla/orangutan
dyad*	name of the two participants that compose the dyad
Session	set of trials - mostly occurring on the same day - usually numerical
Trial	presentation of the experiment to the participants - usually numerical
species_subgroup	species subgroup at time of data collection – WKPRC chimpanzee group a or b/ etc
drop_out	participant drop out /not included in analyses

*This information is only present for dyadic studies.

In addition to the study-specific files contained within each publication folder, the repository also includes three more folders with several files that provide context and support the use of the dataset as a whole.

In the general_files folder:

- Great ape information file (apes_list.csv): Contains general information about the great apes housed at the WKPRC, as provided by WKPRC staff (e.g., name, species, sex, breeding, place of birth, time of arrival to the zoo).
- Domain definition file (domains_definitions.csv): Definitions for cognitive and general domains used in the dataset.
- Experiment list (experiments_table.csv): Details all experiments included in the contributed studies. This file indicates which experiments have available data, participant drop-out information, group IDs, data collection dates, and the ages of participants at the time of testing.
- Frequency of participation by domain file (frequency_participation_domain.csv): Table showing the number of studies each participant has taken part in by domain, with the first study highlighted for each general domain.
- Group ID definitions (group_id_table.csv): Provides definitions for group IDs, including research institution locations and participant species.
- Participation by domain file (participation_social_domain.csv and participation_physical_domain.csv) Chronological lists of studies by cognitive, general domain and participant.
- Domain classification file (study_domains_list.csv): Lists the cognitive, general, and specific domains explored across all contributed studies.
- Contributed studies list (study_table.csv): A compiled list of all contributed studies, including full reference information and published abstracts.

In the general_files/sqlite_dataset folder:

- Database file created using SQLite^194^ (SQLite_database/EVApeCognition.db): Facilitates searching through and navigation of the repository files.
- SQLite example file (SQLite_database/sqlite_examples.ipynb).
- Jupyter notebook with SQLite example queries which can be adapted by users.
- README file: Offers guidance on how to navigate and use the dataset, including file structure, content descriptions, and suggestions for citation.

In the data_standardization_scripts folder:

- Jupyter notebooks used to standardize the originally contributed data files (not included in the repository)^195^.

These files ensure transparency, facilitate reproducibility, and allow users to effectively interpret and navigate the EVApeCognition Dataset.

## Data Overview

**Fig. 3:**
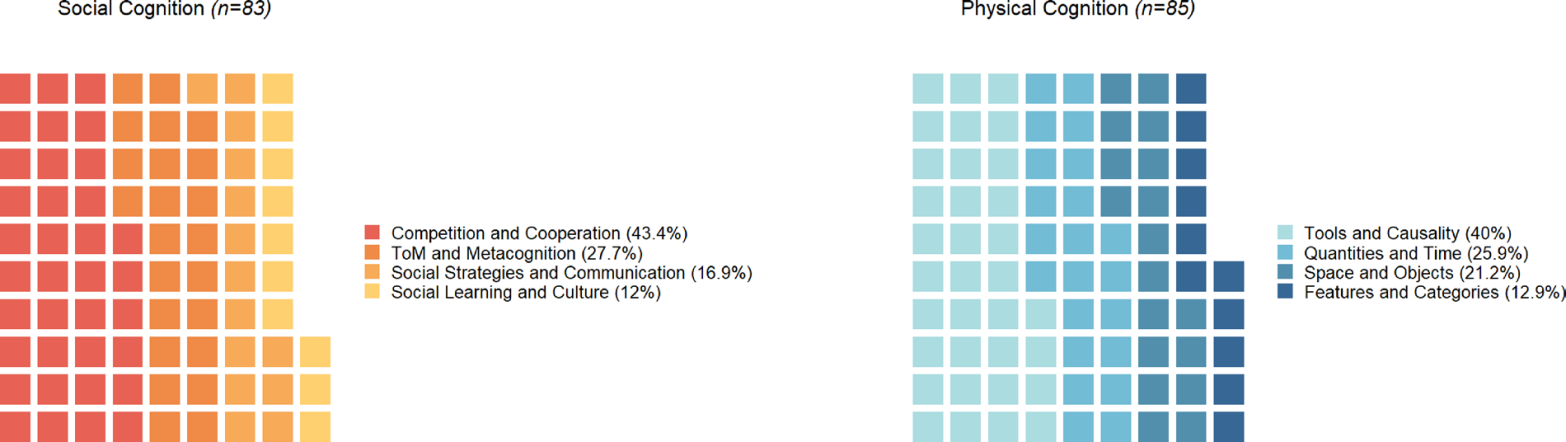
Social and Physical Cognition domains present in the EVApeCognition Dataset. Each study is categorized by one to three pairs of general cognitive domains (hence n = 168, with three additional studies categorized separately and not represented in the plot). More information on cognitive domains is included in the metadata file for each study. See also study_domains_list.csv and domains_definitions.csv in https://github.com/ccp-eva/EVApeCognition for a detailed overview and definitions.

The 150 published studies included in the EVApeCognition Dataset investigated several domains relevant to the study of great ape cognition (Fig. 3). See general_domains_definitions.csv in the repository for more details on these cognitive domains. A total of 81 individual apes participated in these studies between 2001 and 2019. Apes’ participation rates per study varied, ranging from 1 to 122, with an average of almost 40 published studies per ape.

## Technical Validation

### Original data

Corresponding authors interested in contributing their data were provided access to an internal archive maintained by the WKPRC and the Department of Comparative Cultural Psychology at the MPI-EVA since 2008. These archives were created by the corresponding authors or other researchers participating in data collection, with the assistance of WKPRC staff. Original data were added to the relevant archive folder by the corresponding authors during their time at the WKPRC or shortly thereafter for visiting scholars. If their data initially had not been uploaded to the internal archive or a subsequently updated version of the data was available, corresponding authors could send us their datasets via email or upload the files to their archive folder.

### Standardization

The standardization process was conducted by C.M. using Python (ver. 3.9.7; e.g., csv, datetime, json, os, re, collections, see^196^). Additional Python packages used included pandas^197^, pyreadstat^198^, and numpy^199^. To create the standardized data available in the dataset, various original data formats (e.g., .numbers, .txt, .tsv, .xlsx, .sav) were converted to .csv if necessary. The following specific variables from each dataset across selected publications were standardized and included in the following order: study_id, experiment, experiment name, year/month/day of data collection, participant (e.g., name of great ape), age, sex, role, species, session, trial, species_subgroup, and drop_out. If age was provided in a format other than years (e.g., days), an additional variable (i.e., age_in_years) was created to allow for comparison across studies while preserving the original values in case they were relevant to analyses. In the case of dyad and group studies (i.e., 2 + great ape participants), variables associated with the additional participants(s) were also standardized (e.g., participant_2, age_in_years_2, sex_2, role_2, dyad). See Table 1.

All participant information was standardized (e.g., spelling of participant name, sex, species) and relied on the data provided and maintained for accuracy by the staff at the WKPRC. Additional participant information, such as age at the time of participation, was included in individual data sets if provided by the corresponding authors in the dataset itself via subject lists provided in the publication, or if it was possible to calculate using the date of data collection and participant date of birth. If variables intended to be standardized were missing (e.g., day of data collection), this information was not included in the data set unless it was provided by the corresponding author of the relevant publication or it was found through the review of the published manuscript. The rest of the raw data specific to each experiment within each publication are included in each data set after the standardized variables. Characters were replaced to conform to csv standards as needed. UTF-8 encoding was used for experimental data sets and their corresponding glossaries.

All human participants’ data were removed before inclusion of ape data in the dataset. Data from great ape participants who were not currently living at the WKPRC, in addition to data from other species, were included or removed from standardized datasets at the discretion of the corresponding authors, and datasets with excluded participants were labeled accordingly in the metadata. Information regarding name, species, or age of non-WKPRC or non-great ape participants were not added or included in the standardized variables if they were not provided in the original data set or via publication subject lists. Contributed original data sets were organized and separated by experiment within each publication when possible. Data standardization scripts are provided in a subfolder within general_files.

#### Internal review

Internal reviews were conducted to compare the variables mentioned in the publication and those provided in the corresponding dataset(s), verify the standardization of the datasets, and identify any points of clarification needed. A.S. conducted the internal reviews with the assistance of S.M.L-P., S.EvW., C.M., L.M., and A.A.

Following the internal review, the corresponding authors were contacted to answer questions aimed at clarifying the data generated during the internal review process. Example questions could include clarifications about the meaning of specific columns, how to separate datasets by experiment, or the nature of certain variables.

If answers provided by corresponding authors required updates to the standardized data sets, this triggered an additional round of standardization and internal review. This process lasted until the corresponding authors provided approval of the standardized data sets from their publications.

Once the data were fully recovered, approved by the corresponding authors, and incorporated into the dataset, the corresponding authors were asked to fill out a survey to include their name and affiliation, making them co-authors in the dataset publication. Importantly, we allowed corresponding authors to circulate the survey with other co-authors from their contributed publications. The idea was to allow corresponding authors to recognize the collaborative work of their colleagues. Eventually, all corresponding authors and co-authors who completed the survey were included in this publication as co-authors.

#### Data recovery

We received data from 179 of 246 publications, from which we were able to fully standardize and internally review data for 150 (262 experimental datasets, 61% of published studies recovered) after resolving any questions with the corresponding authors by March 31, 2025. For the remaining 29 publications, we were not able to finalize the standardization process due to unresolved questions pending clarification. Figure 2 visualizes step-by-step the process of data recovery. Each publication was classified across a continuum from “total sample” to “final sample”. Publications categorized as “authors contacted” include all those for which we sought data, either via email or in person. “Reply Received” refers to publications for which the corresponding author acknowledged our request for data. “Material Received” includes publications for which the corresponding author provided materials relevant to the publication or directed us to its location, such as within the WKPRC archive or an online repository. Finally, “Dataset standardized” refers to datasets that were standardized after all questions were answered and that are now available in the repository. The repository is meant to continue growing in the future with the inclusion of relevant studies.

## Supplementary information

Supplementary information

## Data Availability

The EVApeCognition Dataset is open-access (CC-BY 4.0) and can be accessed here: 10.5281/zenodo.18846348. Descriptive plots can be accessed here https://osf.io/qm9hd. The lead authors A.S, S.EvW, C.M, A.A, L.M, S.L-P, B.B, L.M, M.T, K.L, J.C and D.H do not take responsibility for the accuracy or completeness of the data provided by corresponding authors in the EVApeCognition Dataset. The corresponding authors of the publications are responsible for the accuracy and completeness of the contributed data from their publications as they accepted the final version for each dataset contained in the EVApeCognition Dataset by the time of publication.

## Code availability

The dataset file created with SQLite Dataset can be accessed here: https://github.com/ccp-eva/EVApeCognition/tree/main/general_files/sqlite_dataset. Python custom code files used to standardize original datasets can be found here: https://github.com/ccp-eva/EVApeCognition/tree/main/data_standardization_scripts. We did not upload original data to the repository to avoid confusion with the standardized files.
