# Supplementary material for: EVApeCognition: An 18-Year Dataset of Great Ape Cognition
Source: Sci Data. 2026 Apr 9;13:805. doi: 10.1038/s41597-026-07191-6 (PMC13223263; doi:10.1038/s41597-026-07191-6)
Supplement: Supplementary file 1 — Supplementary information [file 41597_2026_7191_MOESM1_ESM.docx]

Supplementary Materials for

Title: EVApeCognition: An 18-Year Database Reveals Domain-Specific Characteristics in Great Ape Cognition

**Authors:** Alejandro Sanchez-Amaro^1,2*^, Sonja J. Ebel van Wijk^2,3^, Carin Molenaar^2^, Akzira Abuova^2^, Lizbeth Mujica-Manrique^2^, Sarah M. Leisterer-Peoples^2^, Bret Beheim^4^, Luke Maurits^2^, Anna Albiach-Serrano^8^, Matthias Allritz^2^, Nazli Altınok^9^, Federica Amici^2,3^, Alice MI Auersperg^10^, Filippo Aureli^11, 12^, Elisa Bandini^13,14^, Jochen Barth^15^, Leïla Benziad^6^, Bettina E. Bläsing^16^, Manuel Bohn^2,17^, Marie Bourjade^18^, Juliane Bräuer^19,20^, Marie-Hélène Broihanne^21^, Sarah F. Brosnan^22,^ Nereida Bueno-Guerra^23^, Thomas Bugnyar^81,82^, David Buttelmann^24^, Frances Buttelmann^78^, Trix Cacchione^25^, Malinda Carpenter^7^, Fernando Colmenares^26^, Catherine Crockford^27^, Katherine A. Cronin^28^, África de las Heras^6^, Arianna De Marco^29,30^, Sarah E. DeTroy^2^, Valérie Dufour^31^, Shona Duguid^32^, Robin I. M. Dunbar^33^, Johanna Eckert^34^, Jan M. Engelmann^35^, Joel Fagot^52,53^, Julia Fischer^36, 80^, Sofia Ingrid Fredrika Forss^13^, Martina Funk^37^, György Gergely^38^, Julia R. Greenberg^39^, Johannes Großmann^6^, Sebastian Grüneisen^40^, Marta Halina^41^, Daniel Hanus^2^, Sarah R. Heilbronner^42^, Christophe Heintz^38^, Robert Hepach^33^, Esther Herrmann^43^, Satoshi Hirata^44^, Alenka Hribar^6^, Gabriele Janzen^45,46^, Juliane Kaminski^43^, Patricia Kanngiesser ^77^, Fumihiro Kano^47^, Katharina C. Kirchhofer^6^, Hagen Knofe^6^, Kathrin S. Kopp^2,3^, Christopher Krupenye^48^, Isabelle Barbara Laumer^49^, Stephen C. Levinson^50^, Ulf Liszkowski^51^, Héctor M. Manrique^54,83^, Gema Martin-Ordas^1^, Emma Suvi McEwen^7^, Richard T. Moore^55^, Enric Munar^56^, Marcos Nadal^56^, Christian Nawroth^57^, Suska Nolte^6,7^, Marie Pelé^58^, Patrizia Potì^59^, Hannes Rakoczy^60^, Julia Riedel^61^, Amélie Romain^62^, Federico Rossano^63^, Yvan I. Russell^64^, Gloria Sabbatini^59^, Marie Schäfer^65^, Marina Scheumann^79^, Martin Schmelz^6^, Benjamin Schmid^84^,Vanesa Schmitt^36^, Carla Sebastián-Enesco^66^, Amanda Madeleine Seed^7^, Chikako Suda-King^67^, Tibor Tauzin^68^, Sebastian Tempelmann^69^, Claudio Tennie^70^, Valentina Truppa^59^, Jana Uher^71^, Amrisha Vaish^72^, Edwin van Leeuwen^2,73^, Elisabetta M. Visalberghi^59^, Christoph J. Völter^2,10^, Victoria Vonau^6^, Claudia A.F. Wascher^74^, Roman M. Wittig^27,75^, Wouter Wolf^76^, Michael Tomasello^5,6^, Katja Liebal^2,3^, Josep Call^7^, Daniel B. M. Haun^2*^

*Corresponding author. Email: [alejandro.sanchezamaro@stir.ac.uk](mailto:alejandro.sanchezamaro@stir.ac.uk); [haun@eva.mpg.de](mailto:haun@eva.mpg.de)

**The PDF file includes:**

Supplementary Text

Table S1

Figure S1

**Supplementary Text**

**Table S1. List of 150 studies included in the EVApeCognition Database by the time of publication**

| study_id | year | reference |
| --- | --- | --- |
| albiach2010great | 2010 | Albiach-Serrano, A., Call, J., & Barth, J. (2010). Great apes track hidden objects after changes in the objects' position and in subject's orientation. American Journal of Primatology, 72(4), 349-359. doi:10.1002/ajp.20790. |
| albiach2012apes | 2012 | Albiach-Serrano, A., Bugnyar, T., & Call, J. (2012). Apes (Gorilla gorilla, Pan paniscus, P. troglodytes, Pongo abelii) versus corvids (Corvus corax, C. corone) in a support task: The effect of pattern and functionality. Journal of Comparative Psychology, 126(4), 355-367. doi:10.1037/a0028050. |
| albiach2014reversed | 2014 | Albiach-Serrano, A., & Call, J. (2014). A reversed-reward contingency task reveals causal knowledge in chimpanzees (Pan troglodytes). Animal Cognition, 17(5), 1167-1176. doi:10.1007/s10071-014-0749-9. |
| albiach2015comparing | 2015 | Albiach-Serrano, A., Sebastián-Enesco, C., Seed, A., Colmenares, F., & Call, J. (2015). Comparing humans and nonhuman great apes in the broken cloth problem: Is their knowledge causal or perceptual? Journal of Experimental Child Psychology, 139, 174-189. doi:10.1016/j.jecp.2015.06.004. |
| allritz2013food | 2013 | Allritz, M., Tennie, C., & Call, J. (2013). Food washing and placer mining in captive great apes. Primates, 54(4), 361-370. doi:10.1007/s10329-013-0355-5. |
| allritz2021chimpanzees | 2021 | Allritz, M., McEwen, E. S., & Call, J. (2021). Chimpanzees (Pan troglodytes) show subtle signs of uncertainty when choices are more difficult. Cognition, 214. doi:10.1016/j.cognition.2021.104766. |
| amici2008fission | 2008 | Amici, F., Aureli, F., & Call, J. (2008). Fission-fusion dynamics, behavioral flexibility, and inhibitory control in primates. Current Biology, 18(18), 1415-1419. doi:10.1016/j.cub.2008.08.020. |
| amici2010monkeys | 2010 | Amici, F., Aureli, F., & Call, J. (2010). Monkeys and apes: Are their cognitive skills really so different? American Journal of Physical Anthropology, 143(2), 188-197. doi:10.1002/ajpa.21305. |
| amici2012aversion | 2012 | Amici, F., Call, J., & Aureli, F. (2012). Aversion to violation of expectations of food distribution: the role of social tolerance and relative dominance in seven primate species. Behaviour, 149(3-4), 345-368. doi:10.1163/156853912X637833. |
| amici2012modular | 2012 | Amici, F., Barney, B., Johnson, V. E., Call, J., & Aureli, F. (2012). A modular mind? A test using individual data from seven primate species. PLoS One, 7(12): e51918. doi:10.1371/journal.pone.0051918. |
| amici2014calculated | 2014 | Amici, F., Aureli, F., Mundry, R., Sanchez-Amaro, A., Barroso, A. M., Ferretti, J., & Call, J. (2014). Calculated reciprocity? A comparative test with six primate species. Primates, 55(3), 447-457. doi:10.1007/s10329-014-0424-4. |
| amici2014lack | 2014 | Amici, F., Visalberghi, E., & Call, J. (2014). Lack of prosociality in great apes, capuchin monkeys and spider monkeys: Convergent evidence from two different food distribution tasks. Proceedings of the Royal Society B: Biological Sciences, 281(1793): 20141699. doi:10.1098/rspb.2014.1699. |
| amici2014response | 2014 | Amici, F., Aureli, F., & Call, J. (2014). Response facilitation in the four great apes: Is there a role for empathy? Primates, 55(1), 113-118. doi:10.1007/s10329-013-0375-1. |
| amici2018social | 2018 | Amici, F., Call, J., Watzek, J., Brosnan, S., & Aureli, F. (2018). Social inhibition and behavioural flexibility when the context changes: A comparison across six primate species. Scientific Reports, 8: 3067. doi:10.1038/s41598-018-21496-6. |
| bandini2021individually | 2021 | Bandini, E., Großmann, J., Funk, M., Albiach-Serrano, A., & Tennie, C. (2021). Naïve orangutans (Pongo abelii and Pongo pygmaeus) individually acquire nut-cracking using hammer tools. American Journal of Primatology, 83(9): e23304. doi:10.1002/ajp.23304. |
| barth2006tracking | 2006 | Barth, J., & Call, J. (2006). Tracking the Displacement of Objects: A Series of Tasks With Great Apes (Pan troglodytes, Pan paniscus, Gorilla gorilla, and Pongo pygmaeus) and Young Children (Homo sapiens). Journal of Experimental Psychology: Animal Behavior Processes, 32(3), 239-252. doi:10.1037/0097-7403.32.3.239. |
| bohn2015communication | 2015 | Bohn, M., Call, J., & Tomasello, M. (2015). Communication about absent entities in great apes and human infants. Cognition, 145, 63-72. doi:10.1016/j.cognition.2015.08.009. |
| bohn2016comprehension | 2016 | Bohn, M., Call, J., & Tomasello, M. (2016). Comprehension of iconic gestures by chimpanzees and human children. Journal of Experimental Child Psychology, 142, 1-17. doi:10.1016/j.jecp.2015.09.001. |
| bohn2016role | 2016 | Bohn, M., Call, J., & Tomasello, M. (2016). The role of past interactions in great apes' communication about absent entities. Journal of Comparative Psychology, 130(4), 351-357. doi:10.1037/com0000042. |
| bohn2017information | 2017 | Bohn, M., Allritz, M., Call, J., & Völter, C. J. (2017). Information seeking about tool properties in great apes. Scientific Reports, 7: 10923. doi:10.1038/s41598-017-11400-z. |
| bohn2019natural | 2019 | Bohn, M., Call, J., & Tomasello, M. (2019). Natural reference: A phylo- and ontogenetic perspective on the comprehension of iconic gestures and vocalizations. Developmental Science, 22(2): e12757. doi:10.1111/desc.12757. |
| bohn2020learning | 2020 | Bohn, M., Kordt, C., Braun, M., Call, J., & Tomasello, M. (2020). Learning novel skills from iconic gestures: A developmental and evolutionary perspective. Psychological Science,31(7), 873-880. |
| bourjade2014bonobos | 2014 | Bourjade, M., Call, J., Pelé, M., Maumy, M., & Dufour, V. (2014). Bonobos and orangutans, but not chimpanzees, flexibly plan for the future in a token-exchange task. Animal Cognition, 17(6), 1329-1340. doi:10.1007/s10071-014-0768-6. |
| brauer2005all | 2005 | Bräuer, J., Call, J., & Tomasello, M. (2005). All great ape species follow gaze to distant locations and around barriers. Journal of Comparative Psychology, 119(2), 145-154. doi:10.1037/0735-7036.119.2.145. |
| brauer2006apes | 2006 | Bräuer, J., Call, J., & Tomasello, M. (2006). Are apes really inequity averse? Proceedings of the Royal Society of London / Series B, 273(1605), 3123-3128. doi:10.1098/rspb.2006.3693. |
| brauer2006making | 2006 | Bräuer, J., Kaminski, J., Riedel, J., Call, J., & Tomasello, M. (2006). Making inferences about the location of hidden food: Social dog - casual ape. Journal of Comparative Psychology, 120(1), 38-47. doi:10.1037/0735-7036.120.1.38. |
| brauer2007chimpanzees | 2007* | Bräuer, J., Call, J., & Tomasello, M. (2007). Chimpanzees Really Know What Others Can See in a Competitive Situation. Animal Cognition, 10(4), 439-448. doi:10.1007/s10071-007-0088-1. |
| brauer2008chimpanzees | 2008 | Bräuer, J., Call, J., & Tomasello, M. (2008). Chimpanzees do not take into account what others can hear in a competitive situation. Animal Cognition, 11(1), 175-178. doi:10.1007/s10071-007-0097-0. |
| brauer2009apes | 2009 | Bräuer, J., Call, J., & Tomasello, M. (2009). Are apes inequity averse? New data on the token-exchange paradigm. American Journal of Primatology, 71(2), 175-181. doi:10.1002/ajp.20639. |
| brauer2015apes | 2015 | Bräuer, J., & Call, J. (2015). Apes produce tools for future use. American Journal of Primatology, 77(3), 254-263. doi:10.1002/ajp.22341. |
| broihanne2019monkeys | 2019 | Broihanne, M.-H., Romain, A., Call, J., Thierry, B., Wascher, C. A. F., De Marco, A., Verrier, D., & Dufour, V. (2019). Monkeys (Sapajus apella and Macaca tonkeana) and great apes (Gorilla gorilla, Pongo abelii, Pan paniscus, and Pan troglodytes) play for the highest bid. Journal of Comparative Psychology, 133(3), 301-312. doi:10.1037/com0000153. |
| bueno2019bargaining | 2019 | Bueno-Guerra, N., Völter, C. J., de las Heras, A., Colell, M., & Call, J. (2019). Bargaining in chimpanzees (Pan troglodytes): The effect of cost, emount of gift, reciprocity, and communication (advance online). Journal of Comparative Psychology. doi:10.1037/com0000189. |
| bueno2020effects | 2020 | Bueno-Guerra, N., Colell, M., & Call, J. (2020). Effects of indirect reputation and type of rearing on food choices in chimpanzees (Pan troglodytes). Behavioral Ecology and Sociobiology, 74: 79. doi:10.1007/s00265-020-02861-w. |
| buttelmann2008behavioral | 2008 | Buttelmann, D., Call, J., & Tomasello, M. (2008). Behavioral cues that great apes use to forage for hidden food. Animal Cognition, 11(1), 117-128. doi:10.1007/s10071-007-0095-2. |
| buttelmann2017great | 2017 | Buttelmann, D., Buttelmann, F., Carpenter, M., Call, J., & Tomasello, M. (2017). Great apes distinguish true from false beliefs in an interactive helping task. PLoS One, 12(4): e0173793. doi:10.1371/journal.pone.0173793. |
| cacchione2009gravity | 2009 | Cacchione, T., Call, J., & Zingg, R. (2009). Gravity and Solidity in Four Great Ape Species (Gorilla gorilla, Pongo pygmaeus, Pan troglodytes, Pan paniscus): Vertical and Horizontal Variations of the Table Task. Journal of Comparative Psychology, 123(2), 168-180. doi:10.1037/a0013580. |
| cacchione2014apes | 2014 | Cacchione, T., Hrubesch, C., & Call, J. (2014). Apes' tracking of objects and collections. Swiss Journal of Psychology, 73(1), 47-52. doi:10.1024/1421-0185/a000120. |
| call2004inferences | 2004 | Call, J. (2004). Inferences About the Location of Food in the Great Apes (Pan panicus, Pan troglodytes, Gorilla gorilla, and Pongo pygmaeus). Journal of Comparative Psychology, 118(2), 232-241. doi:10.1037/0735-7036.118.2.232. |
| dufour2009calculated | 2009 | Dufour, V., Péle, M., Neumann, M., Thierry, B., & Call, J. (2009). Calculated reciprocity after all: computation behind token transfers in orang-utans. Biology Letters, 5(2), 172-175. doi:10.1098/rsbl.2008.0644. |
| duguid2014coordination | 2014 | Duguid, S., Wyman, E., Bullinger, A. F., Herfurth-Majstorovic, K., & Tomasello, M. (2014). Coordination strategies of chimpanzees and human children in a Stag Hunt game. Proceedings of the Royal Society B: Biological Sciences, 281(1796): 20141973. doi:10.1098/rspb.2014.1973. |
| duguid2020strategies | 2020 | Duguid, S., Wyman, E., Grueneisen, S., & Tomasello, M. (2020). The strategies used by chimpanzees (Pan troglodytes) and children (Homo sapiens) to solve a simple coordination problem. Journal of Comparative Psychology, 134(4), 401-411. doi:10.1037/com0000220. |
| ebel2019innovative | 2019 | Ebel, S. J., Schmelz, M., Herrmann, E., & Call, J. (2019). Innovative problem solving in great apes: The role of visual feedback in the floating peanut task. Animal Cognition, 22(5), 791-805. doi:10.1007/s10071-019-01275-0. |
| ebel2020object | 2020 | Ebel, S. J., Kopp, K. S., & Liebal, K. (2020). Object preferences in captive Sumatran orang-utans (Pongo abelii). Behavioural Processes, 170: 103993. doi:10.1016/j.beproc.2019.103993. |
| ebel2021prior | 2021 | Ebel, S. J., Völter, C. J., & Call, J. (2021). Prior experience mediates the usage of food items as tools in great apes (Pan paniscus, Pan troglodytes, Gorilla gorilla, and Pongo abelii).Journal of Comparative Psychology,135(1), 64-73. |
| eckert2017great | 2017 | Eckert, J., Rakoczy, H., & Call, J. (2017). Are great apes able to reason from multi-item samples to populations of food items? American Journal of Primatology, 79(10): e22693. doi:10.1002/ajp.22693. |
| engelmann2012five | 2012 | Engelmann, J. M., Herrmann, E., & Tomasello, M. (2012). Five-year olds, but not chimpanzees, attempt to manage their reputations. PLoS One, 7(10): e48433. doi:10.1371/journal.pone.0048433. |
| engelmann2017social | 2017 | Engelmann, J. M., Clift, J. B., Herrmann, E., & Tomasello, M. (2017). Social disappointment explains chimpanzees' behaviour in the inequity aversion task. Proceedings of the Royal Society B: Biological Sciences, 284(1861): 20171502. doi:10.1098/rspb.2017.1502. |
| forss2016cognitive | 2016 | Forss, S., Willems, E., Call, J., & van Schaik, C. (2016). Cognitive differences between orang-utan species: a test of the cultural intelligence hypothesis. Scientific Reports, 6, 30516. doi:10.1038/srep30516. |
| greenberg2010chimpanzee | 2010 | Greenberg, J. R., Hamann, K., Warneken, F., & Tomasello, M. (2010). Chimpanzee helping in collaborative and noncollaborative contexts. Animal Behaviour, 80(5), 873-880. doi:10.1016/j.anbehav.2010.08.008. |
| grueneisen2017children | 2017 | Grüneisen, S., Duguid, S., Saur, H., & Tomasello, M. (2017). Children, chimpanzees, and bonobos adjust the visibility of their actions for cooperators and competitors. Scientific Reports, 7: 8504. doi:10.1038/s41598-017-08435-7. |
| halina2018goal | 2018 | Halina, M., Liebal, K., & Tomasello, M. (2018). The goal of ape pointing. PLoS One, 13(4): e0195182. doi:10.1371/journal.pone.0195182. |
| hanus2011chimpanzee | 2011 | Hanus, D., & Call, J. (2011). Chimpanzee problem-solving: contrasting the use of causal and arbitrary cues. Animal Cognition, 14(6), 871-878. doi:10.1007/s10071-011-0421-6. |
| hanus2011comparing | 2011 | Hanus, D., Mendes, N., Tennie, C., & Call, J. (2011). Comparing the performances of apes (Gorilla gorilla, Pan troglodytes, Pongo pygmaeus) and human children (Homo sapiens) in the floating peanut task. PLoS ONE, 6(6): e19555. doi:10.1371/journal.pone.0019555. |
| haun2006evolutionary | 2006 | Haun, D. B. M., Call, J., Janzen, G., & Levinson, S. C. (2006). Evolutionary psychology of spatial representations in the Hominidae. Current Biology, 16(17), 1736-1740. doi:10.1016/j.cub.2006.07.049. |
| haun2009great | 2009 | Haun, D. B. M., & Call, J. (2009). Great apes’ capacities to recognize relational similarity. Cognition, 110(2), 147-159. doi:10.1016/j.cognition.2008.10.012. |
| haun2011great | 2011 | Haun, D. B. M., Nawroth, C., & Call, J. (2011). Great apes' risk-taking strategies in a decision making task. PLoS ONE, 6(12): e28801. doi:10.1371/journal.pone.0028801. |
| heilbronner2008fruit | 2008 | Heilbronner, S. R., Rosati, A. G., Stevens, J. R., Hare, B., & Hauser, M. D. (2008). A fruit in the hand or two in the bush? Divergent risk preferences in chimpanzees and bonobos. Biology Letters, 4(3), 246-249. doi:10.1098/rsbl.2008.0081. |
| hepach2020help | 2020 | Hepach, R., Benziad, L., & Tomasello, M. (2020). Chimpanzees help others with what they want; children help them with what they need. Developmental Science, 23(3): e12922. doi:10.1111/desc.12922. |
| hepach2021chimpanzees | 2021 | Hepach, R., Vaish, A., Kano, F., Benziad, L., Albiach-Serrano, A., Call, J., & Tomasello, M. (2021). Chimpanzees’ (Pan troglodytes) internal arousal remains elevated if they cannot themselves help a conspecific. Journal of Comparative Psychology, 135(2), 196-207. doi:10.1037/com0000255. |
| herrmann2006apes | 2006 | Herrmann, E., & Tomasello, M. (2006). Apes' and children's understanding of cooperative and competitive motives in a communicative situation. Developmental Science, 9(5), 518-529. doi:10.1111/j.1467-7687.2006.00519.x. |
| herrmann2019human | 2019 | Herrmann, E., Haux, L. M., Zeidler, H., & Engelmann, J. M. (2019). Human children but not chimpanzees make irrational decisions driven by social comparison. Proceedings of the Royal Society of London: B, Biological Sciences, 286(1894): 20182228. doi:10.1098/rspb.2018.2228. |
| hribar2011great | 2011 | Hribar, A., & Call, J. (2011). Great apes use landmark cues over spatial relations to find hidden food. Animal Cognition, 14(5), 623-635. doi:10.1007/s10071-011-0397-2. |
| kanngiesser2010bonobos | 2010 | Kanngiesser, P., & Call, J. (2010). Bonobos, chimpanzees, gorillas, and orang utans use feature and spatial cues in two spatial memory tasks. Animal Cognition, 13(3), 419-430. doi:10.1007/s10071-009-0291-3. |
| kanngiesser2011limits | 2011 | Kanngiesser, P., Santos, L. R., Hood, B. M., & Call, J. (2011). The Limits of Endowment Effects in Great Apes (Pan paniscus, Pan troglodytes, Gorilla gorilla, Pongo pygmaeus). Journal of Comparative Psychology, 125(4), 436-445. doi:10.1037/a0024516. |
| kanngiesser2020children | 2020 | Kanngiesser, P., Rossano, F., Frickel, R., Tomm, A., & Tomasello, M. (2020). Children, but not great apes, respect ownership. Developmental Science, 23(1): e12842. doi:10.1111/desc.12842. |
| kano2011visual | 2011 | Kano, F., Hirata, S., Call, J., & Tomonaga, M. (2011). The visual strategy specific to humans among hominids: A study using the gap–overlap paradigm. Vision Research, 51(23-24), 2348-2355. doi:10.1016/j.visres.2011.09.006. |
| kano2012face | 2012 | Kano, F., Call, J., & Tomonaga, M. (2012). Face and eye scanning in gorillas (Gorilla gorilla), orangutans (Pongo abelii), and humans (Homo sapiens): Unique eye-viewing patterns in humans among hominids. Journal of Comparative Psychology, 126(4), 388-398. doi:10.1037/a0029615. |
| kano2014cross | 2014 | Kano, F., & Call, J. (2014). Cross-species variation in gaze following and conspecific preference among great apes, human infants and adults. Animal Behaviour, 91, 137-150. doi:10.1016/j.anbehav.2014.03.011. |
| kano2014great | 2014 | Kano, F., & Call, J. (2014). Great apes generate goal-based action predictions: An eye-tracking study. Psychological Science, 25(9), 1691-1698. doi:10.1177/0956797614536402. |
| kano2015social | 2015 | Kano, F., Hirata, S., & Call, J. (2015). Social attention in the two species of pan: Bonobos make more eye contact than chimpanzees. PLoS One, 10(6): e0129684. doi:10.1371/journal.pone.0129684. OA |
| kano2016nasal | 2016 | Kano, F., Hirata, S., Deschner, T., Behringer, V., & Call, J. (2016). Nasal temperature drop in response to a playback of conspecific fights in chimpanzees: A thermo-imaging study. Physiology & Behavior, 155, 83-94. doi:10.1016/j.physbeh.2015.11.029. |
| kano2018human | 2018 | Kano, F., Moore, R., Krupenye, C., Hirata, S., Tomonaga, M., & Call, J. (2018). Human ostensive signals do not enhance gaze following in chimpanzees, but do enhance object-oriented attention. Animal Cognition, 21(5), 715-728. doi:10.1007/s10071-018-1205-z. |
| kano2018primate | 2018 | Kano, F., Shepherd, S. V., Hirata, S., & Call, J. (2018). Primate social attention: Species differences and effects of individual experience in humans, great apes, and macaques. PLoS One, 13(2): e0193283. doi:10.1371/journal.pone.0193283. |
| kano2019great | 2019 | Kano, F., Krupenye, C., Hirata, S., Tomonaga, M., & Call, J. (2019). Great apes use self-experience to anticipate an agent’s action in a false-belief test. Proceedings of the National Academy of Sciences, 116(42), 20904-20909. doi:10.1073/pnas.1910095116. |
| karg2016differing | 2016 | Karg, K., Schmelz, M., Call, J., & Tomasello, M. (2016). Differing views: Can chimpanzees do Level 2 perspective-taking? Animal Cognition, 19(3), 555-564. doi:10.1007/s10071-016-0956-7. OA |
| kirchhofer2012dogs | 2012 | Kirchhofer, K. C., Zimmermann, F., Kaminski, J., & Tomasello, M. (2012). Dogs (Canis familiaris), but Not Chimpanzees (Pan troglodytes), Understand Imperative Pointing. PLoS One, 7(2): e30913. doi:10.1371/journal.pone.0030913. |
| knofe2019chimpanzees | 2019 | Knofe, H., Engelmann, J., Tomasello, M., & Herrmann, E. (2019). Chimpanzees monopolize and children take turns in a limited resource problem. Scientific Reports, 9: 7597. doi:10.1038/s41598-019-44096-4. |
| kopp2021small | 2021 | Kopp, K. S., Ebel, S. J., Wittig, R. M., Haun, D. B. M., & Crockford, C. (2021). Small mirrors do the trick: A simple, but effective method to study mirror self-recognition in chimpanzees. Animal Behavior and Cognition, 8(3), 391-404. doi:10.26451/abc.08.03.05.2021. |
| krupenye2016great | 2016 | Krupenye, C., Kano, F., Hirata, S., Call, J., & Tomasello, M. (2016). Great apes anticipate that other individuals will act according to false beliefs. Science, 354(6308), 110-114. doi:10.1126/science.aaf8110. |
| krupenye2017test | 2017 | Krupenye, C., Kano, F., Hirata, S., Call, J., & Tomasello, M. (2017). A test of the submentalizing hypothesis: Apes' performance in a false belief task inanimate control. Communicative & Integrative Biology, 10(4): e1343771. doi:10.1080/19420889.2017.1343771. |
| laumer2018spontaneous | 2018 | Laumer, I. B., Call, J., Bugnyar, T., & Auersperg, A. M. I. (2018). Spontaneous innovation of hook-bending and unbending in orangutans (Pongo abelii). Scientif Reports, 8, 16518. doi:10.1038/s41598-018-34607-0. |
| laumer2019orangutans | 2019 | Laumer, I. B., Auersperg, A. M. I., Bugnyar, T., & Call, J. (2019). Orangutans (Pongo abelii) make flexible decisions relative to reward quality and tool functionality in a multi-dimensional tool-use task. PLoS One, 14(2): e0211031. doi:10.1371/journal.pone.0211031. |
| leeuwen2013total | 2013 | Van Leeuwen, E. J. C., Cronin, K. A., Schütte, S., Call, J., & Haun, D. B. M. (2013). Chimpanzees (Pan troglodytes) flexibly adjust their behaviour in order to maximize payoffs, not to conform to majorities. PLoS One, 8(11): e80945. doi:10.1371/journal.pone.0080945. |
| leeuwen2014human | 2014 | Van Leeuwen, E. J. C., Call, J., & Haun, D. B. M. (2014). Human children rely more on social information than chimpanzees do. Biology Letters, 10(11): 20140487. doi:10.1098/rsbl.2014.0487. |
| leeuwen2017conservatism | 2017 | Van Leeuwen, E. J. C., & Call, J. (2017). Conservatism and 'copy-if-better' in chimpanzees (Pan troglodytes). Animal Cognition, 20(3), 575-579. doi:10.1007/s10071-016-1061-7. |
| lewis2017non | 2017 | Lewis, A. V. M., Call, J., & Berntsen, D. (2017). Non-goal-directed recall of specific events in apes after long delays. Proceedings of the Royal Society of London. Series B: Biological Sciences, 284(1858): 20170518. doi:10.1098/rspb.2017.0518. |
| liebal2014does | 2014 | Liebal, K., Vaish, A., Haun, D., & Tomasello, M. (2014). Does sympathy motivate prosocial behaviour in great apes? PLoS One, 9(1): e84299. doi:doi:10.1371/journal.pone.0084299. OA |
| liebal2017give | 2017 | Liebal, K., & Rossano, F. (2017). The give and take of food sharing in Sumatran orang-utans, Pongo abelii, and chimpanzees, Pan troglodytes. Animal Behaviour, 133, 91-100. doi:10.1016/j.anbehav.2017.09.006. |
| liszkowski2009prelinguistic | 2009 | Liszkowski, U., Schäfer, M., Carpenter, M., & Tomasello, M. (2009). Prelinguistic infants, but not chimpanzees, communicate about absent entities. Psychological Science, 20(5), 654-660. |
| manrique2010great | 2010 | Manrique, H. M., Gross, A.-N.-M., & Call, J. (2010). Great Apes Select Tools on the Basis of Their Rigidity. Journal of Experimental Psychology: Animal Behavior Processes, 36(4), 409-422. doi:10.1037/a0019296. |
| manrique2011spontaneous | 2011 | Manrique, H. M., & Call, J. (2011). Spontaneous use of tools as straws in great apes. Animal Cognition, 14(2), 213-226. doi:10.1007/s10071-010-0355-4. |
| manrique2013repeated | 2013 | Manrique, H. M., Völter, C. J., & Call, J. (2013). Repeated innovation in great apes. Animal Behaviour, 85(1), 195-202. doi:10.1016/j.anbehav.2012.10.026. |
| manrique2015age | 2015 | Manrique, H. M., & Call, J. (2015). Age-dependent cognitive inflexibility in great apes. Animal Behaviour, 102, 1-6. doi:10.1016/j.anbehav.2015.01.002. |
| many2019establishing | 2019 | Many Primates, Altschul, D. M., Beran, M. J., Bohn, M., Call, J., DeTroy, S., Duguid, S. J., Egelkamp, C. L., Fichtel, C., Fischer, J., Flessert, M., Hanus, D., Haun, D. B. M., Haux, L. M., Hernandez-Aguilar, R. A., Herrmann, E., Hopper, L. M., Joly, M., Kano, F., Keupp, S., Melis, A. P., Motes Rodrigo, A., Ross, S. R., Sánchez Amaro, A., Sato, Y., Schmitt, V., Schweinfurth, M. K., Seed, A. M., Taylor, D., Völter, C. J., Warren, E., & Watzek, J. (2019). Establishing an infrastructure for collaboration in primate cognition research. PLoS One, 14(10): e0223675. doi:10.1371/journal.pone.0223675. |
| martin2008tubes | 2008 | Martin-Ordas, G., Call, J., & Colmenares, F. (2008). Tubes, tables and traps: great apes solve two functionally equivalent trap tasks but show no evidence of transfer across tasks. Animal Cognition, 11(3), 423-430. doi:10.1007/s10071-007-0132-1. |
| martin2009assessing | 2009 | Martin-Ordas, G., & Call, J. (2009). Assessing Generalization Within and Between Trap Tasks in the Great Apes. International Journal of Comparative Psychology, 22(1), 43-60. |
| martin2010keeping | 2010 | Martin-Ordas, G., Haun, D. B. M., Colmenares, F., & Call, J. (2010). Keeping track of time: Evidence for episodic-like memory in great apes. Animal Cognition, 13(2), 331-340. doi:10.1007/s10071-009-0282-4. |
| martin2011memory | 2011 | Martin-Ordas, G., & Call, J. (2011). Memory processing in great apes: the effect of time and sleep. Biology Letters, 7(6), 829-832. doi:10.1098/rsbl.2011.0437. |
| martin2013memory | 2013 | Martin-Ordas, G., Berntsen, D., & Call, J. (2013). Memory for distant past events in chimpanzees and orangutans. Current Biology, 23(15), 1438-1441. doi:10.1016/j.cub.2013.06.017. |
| moore2015production | 2015 | Moore, R., Call, J., & Tomasello, M. (2015). Production and comprehension of gestures between orang-utans (Pongo pygmaeus) in a referential communication game. PLoS One, 10(6): e0129726. doi:10.1371/journal.pone.0129726. OA |
| muhlenbeck2015gaze | 2015 | Mühlenbeck, C., Liebal, K., Pritsch, C., & Jacobsen, T. (2015). Gaze duration biases for colours in combination with dissonant and consonant sounds: a comparative eye-tracking study with orangutans. PLoS One, 10(10), e0139894. |
| muhlenbeck2016differences | 2016 | Mühlenbeck, C., Liebal, K., Pritsch, C., & Jacobsen, T. (2016). Differences in the visual perception of symmetric patterns in orangutans (Pongo pygmaeus abelii) and two human cultural groups: A comparative eye-tracking study. Frontiers in Psychology, 7, 408. |
| muhlenbeck2017cultural | 2017 | Mühlenbeck, C., Jacobsen, T., Pritsch, C., & Liebal, K. (2017). Cultural and species differences in gazing patterns for marked and decorated objects: a comparative eye-tracking study. Frontiers in psychology, 8, 6. |
| munar2015common | 2015 | Munar, E., Gómez-Puerto, G., Call, J., & Nadal, M. (2015). Common visual preference for curved contours in humans and great apes. PLoS One, 10(11): e0141106. doi:10.1371/journal.pone.0141106. |
| nolte2021targeted | 2021 | Nolte, S., & Call, J. (2021). Targeted helping and cooperation in zoo-living chimpanzees and bonobos. Royal Society Open Science, 8: 201688. doi:10.1098/rsos.201688. |
| parron2008behavioural | 2008 | Parron, C., Call, J., & Fagot, J. (2008). Behavioural responses to photographs by pictorially naïve baboons (Papio anubis), gorillas (Gorilla gorilla) and chimpanzees (Pan troglodytes). Behavioural Processes, 78(3), 351-357. doi:10.1016/j.beproc.2008.01.019. |
| poti2010searching | 2010 | Potì, P., Kanngiesser, P., Saporiti, M., Amiconi, A., Bläsing, B., & Call, J. (2010). Searching in the Middle—Capuchins' (Cebus apella) and Bonobos' (Pan paniscus) Behavior During a Spatial Search Task. Journal of Experimental Psychology: Animal Behavior Processes, 36(1), 92-109. doi:10.1037/a0015970. |
| rakoczy2014apes | 2014 | Rakoczy, H., Clüver, A., Saucke, L., Stoffregen, N., Gräbener, A., Migura, J., & Call, J. (2014). Apes are intuitive statisticians. Cognition, 131(1), 60-68. doi:10.1016/j.cognition.2013.12.011. |
| romain2021non | 2021 | Romain, A., Broihanne, M.-H., De Marco, A., Ngoubangoye, B., Call, J., Rebout, N., & Dufour, V. (2021). Non-human primates use combined rules when deciding under ambiguity. Philosophical Transactions of the Royal Society B: Biological Sciences, 376(1819): 20190672. doi:10.1098/rstb.2019.0672. |
| russell2008image | 2008 | Russell, Y. I, Call, J., & Dunbar, R. I. M. (2008). Image scoring in great apes. Behavioural Processes, 78(1), 108-111. doi:10.1016/j.beproc.2007.10.009. |
| sabbatini2012understanding | 2012 | Sabbatini, G., Truppa, V., Hribar, A., Gambetta, B., Call, J., & Visalberghi, E. (2012). Understanding the functional properties of tools: chimpanzees (Pan troglodytes) and capuchin monkeys (Cebus apella) attend to tool features differently. Animal Cognition, 15(4), 577-590. doi:10.1007/s10071-012-0486-x. |
| sanchez2016chimpanzees | 2016 | Sanchez-Amaro, A., Duguid, S., Call, J., & Tomasello, M. (2016). Chimpanzees coordinate in a snowdrift game. Animal Behaviour, 116, 61-74. doi:10.1016/j.anbehav.2016.03.030. |
| sanchez2016differences | 2016 | Sanchez-Amaro, A., Pereto, M., & Call, J. (2016). Differences in between-reinforcer value modulate the selective-value effect in great apes (Pan Troglodyes, P. Paniscus, Gorilla Gorilla, Pongo Abelii). Journal of Comparative Psychology, 130(1), 1-12. doi:10.1037/com0000014. |
| sanchez2017chimpanzees | 2017 | Sanchez-Amaro, A., Duguid, S., Call, J., & Tomasello, M. (2017). Chimpanzees, bonobos and children successfully coordinate in conflict situations. Proceedings of the Royal Society B: Biological Sciences, 284(1856): 20170259. doi:10.1098/rspb.2017.0259. |
| sanchez2018chimpanzees | 2018 | Sanchez-Amaro, A., Duguid, S., Call, J., & Tomasello, M. (2018). Chimpanzees’ understanding of social leverage. PLoS One, 13(12): e0207868. doi:10.1371/journal.pone.0207868. |
| sanchez2019chimpanzees | 2019 | Sanchez-Amaro, A., Duguid, S., Call, J., & Tomasello, M. (2019). Chimpanzees and children avoid mutual defection in a social dilemma. Evolution and Human Behavior, 40(1), 46-54. doi:10.1016/j.evolhumbehav.2018.07.004. |
| sanchez2019disentangling | 2019 | Sanchez-Amaro, A., Altinok, N., Heintz, C., & Call, J. (2019). Disentangling great apes’ decoy-effect bias in a food choice task. Animal Behaviour and Cognition, 6 (3), 213-222. doi:10.26451/abc.06.03.05.2019. |
| sanchez2021chimpanzees | 2021 | Sanchez-Amaro, A., & Rossano, F. (2021). Chimpanzees and bonobos use social leverage in an ultimatum Game. Proceedings of the Royal Society B: Biological Sciences, 288 (1962): 20211937. doi:10.1098/rspb.2021.1937. |
| scheumann2006sumatran | 2006 | Scheumann, M., & Call, J. (2006). Sumatran orangutans (Pongo abelii) and a yellow-cheeked crested gibbon (Nomascus gabriellae) know what is where. International Journal of Primatology, 27(2), 575-602. doi:10.1007/s10764-006-9024-5. |
| schmelz2011chimpanzees | 2011 | Schmelz, M., Call, J., & Tomasello, M. (2011). Chimpanzees know that others make inferences. Proceedings of the National Academy of Sciences of the United States of America, 108(7), 3077-3079. doi:10.1073/pnas.1000469108. |
| schmelz2013chimpanzees | 2013 | Schmelz, M., Call, J., & Tomasello, M. (2013). Chimpanzees predict that a competitor’s preference will match their own. Biology Letters, 9(1): 20120829. doi:10.1098/rsbl.2012.0829. |
| schmelz2017chimpanzees | 2017 | Schmelz, M. *., Grueneisen, S. *., Kabalak, A., Jost, J., & Tomasello, M. (2017). Chimpanzees return favors at a personal cost. Proceedings of the National Academy of Sciences of the United States of America, 114(28), 7462-7467. doi:10.1073/pnas.1700351114. |
| schmelz2020psychological | 2020 | Schmelz, M., Grüneisen, S., & Tomasello, M. (2020). The psychological mechanisms underlying reciprocal prosociality in chimpanzees (Pan troglodytes). Journal of Comparative Psychology, 134(2), 149-157. doi:10.1037/com0000200. |
| schmid2017great | 2017 | Schmid, B., Karg, K., Perner, J., & Tomasello, M. (2017). Great apes are sensitive to prior reliability of an informant in a gaze following task. PLoS One, 12(11): e0187451. doi:10.1371/journal.pone.0187451. |
| schmitt2013monkeys | 2013 | Schmitt, V., Kroeger, I., Zinner, D., Call, J., & Fischer, J. (2013). Monkeys perform as well as apes and humans in a size discrimination task. Animal Cognition, 16(5), 829-838. doi:10.1007/s10071-013-0616-0. |
| seed2012chimpanzee | 2012 | Seed, A., Seddon, E., Greene, B., & Call, J. (2012). Chimpanzee 'folk physics': bringing failures into focus. Philosophical Transactions of the Royal Society of London. Series B, Biological Sciences, 367(1603), 2743-2752. doi:10.1098/rstb.2012.0222. |
| suda2004piagetian | 2004 | Suda, C., & Call, J. (2004). Piagetian Liquid Conservation in the Great Apes (Pan paniscus, Pan troglodytes, and Pongo pygmaeus). Journal of Comparative Psychology, 118(3), 265-279. doi:10.1037/0735-7036.118.3.265. |
| suda2005piagetian | 2005 | Suda, C., & Call, J. (2005). Piagetian conservation of discrete quantities in bonobos (Pan paniscus), chimpanzees (Pan troglodytes), and orangutans (Pongo pygmaeus). Animal Cognition, 8(4), 220-235. doi:10.1007/s10071-004-0247-6. |
| suda2006what | 2006 | Suda, C., & Call, J. (2006). What does an intermediate success rate mean? An analysis of a Piagetian liquid conservation task in the great apes. Cognition, 99(1), 53-71. doi:10.1016/j.cognition.2005.01.005. |
| tauzin2020context | 2020 | Tauzin, T., Bohn, M., Gergely, G., & Call, J. (2020). Context-sensitive adjustment of pointing in great apes. Scientific Reports, 10: 1048. doi:10.1038/s41598-019-56183-7. |
| tempelmann2013apes | 2013 | Tempelmann, S., Kaminski, J., & Liebal, K. (2013). When apes point the finger: Three great ape species fail to use a conspecific’s imperative pointing gesture. Interaction studies, 14(1), 7-23. doi:10.1075/is.14.1.02tem. |
| tennie2006push | 2006 | Tennie, C., & Call, J. (2006). Push or pull: emulation versus imitation in great apes and human children. Ethology, 112(12), 1159-1169. doi:10.1111/j.1439-0310.2006.01269.x. |
| tennie2009ratcheting | 2009 | Tennie, C., Call, J., & Tomasello, M. (2009). Ratcheting up the ratchet: on the evolution of cumulative culture. Philosophical Transactions of the Royal Society B, 364(1528), 2405-2415. doi:10.1098/rstb.2009.0052. |
| tennie2010evidence | 2010 | Tennie, C., Call, J., & Tomasello, M. (2010). Evidence for emulation in chimpanzees in social settings using the floating peanut task. PLoS ONE, 5(5): e10544. doi:10.1371/journal.pone.0010544. |
| tennie2010two | 2010 | Tennie, C., Greve, K., Gretscher, H., & Call, J. (2010). Two-year-old children copy more reliably and more often than nonhuman great apes in multiple observational learning tasks. Primates, 51(4), 337-351. doi:10.1007/s10329-010-0208-4. |
| tennie2019chimpanzees | 2019 | Tennie, C., Völter, C. J., Vonau, V., Hanus, D., Call, J., & Tomasello, M. (2019). Chimpanzees use observed temporal directionality to learn novel causal relations (advance online). Primates. doi:10.1007/s10329-019-00754-9. |
| uher2008coherence | 2008 | Uher, J., Asendorpf, J. B., & Call, J. (2008). Personality in the behaviour of great apes: temporal stability, cross-situational consistency and coherence in response. Animal Behaviour, 75(1), 99-112. doi:10.1016/j.anbehav.2007.04.018. |
| uher2008great | 2008 | Uher, J., & Call, J. (2008). How the Great Apes (Pan troglodytes, Pongo pygmaeus, Pan paniscus, Gorilla gorilla) Perform on the Reversed Reward Contingency Task II: Transfer to New Quantities, Long-Term Retention, and the Impact of Quantity Ratios. Journal of Comparative Psychology, 122(2), 204-212. doi:10.1037/0735-7036.122.2.204. |
| uher2008personality | 2008 | Uher, J., & Asendorpf, J. B. (2008). Personality assessment in the Great Apes: Comparing ecologically valid behavior measures, behavior ratings, and adjective ratings. Journal of Research in Personality, 42(4), 821-838. doi:10.1016/j.jrp.2007.10.004. |
| vlamings2006great | 2006 | Vlamings, P., Uher, J., & Call, J. (2006). How the Great apes (Pan troglodytes Pongo pygmaeus, Pan paniscus and Gorilla gorilla) perform on the reversed contingency task: the effects of food quantity and food visibility. Journal of Experimental Psychology: Animal Behavior Processes, 32(1), 60-70. doi:10.1037/0097-7403.32.1.60. |
| volter2012problem | 2012 | Völter, C., & Call, J. (2012). Problem solving in great apes (Pan paniscus, Pan troglodytes, Gorilla gorilla, and Pongo abelii): the effect of visual feedback. Animal Cognition, 15(5), 923-936. doi:10.1007/s10071-012-0519-5. |
| volter2014cognitive | 2014 | Völter, C. J., & Call, J. (2014). The cognitive underpinnings of flexible tool use in great apes. Journal of Experimental Psychology: Animal Learning and Cognition, 40(3), 287-302. doi:10.1037/xan0000025. |
| volter2014great | 2014 | Völter, C. J., & Call, J. (2014). Great apes (Pan paniscus, Pan troglodytes, Gorilla gorilla, Pongo abelii) follow visual trails to locate hidden food. Journal of Comparative Psychology, 128(2), 199-208. doi:10.1037/a0035434. |
| volter2014younger | 2014 | Völter, C. J., & Call, J. (2014). Younger apes and human children plan their moves in a maze task. Cognition, 130(2), 186-203. doi:10.1016/j.cognition.2013.10.007. |
| volter2015exploitation | 2015 | Völter, C. J., Rossano, F., & Call, J. (2015). From exploitation to cooperation: Social tool use in orang-utan mother–offspring dyads. Animal Behaviour, 100, 126-134. doi:10.1016/j.anbehav.2014.11.025. |
| volter2016great | 2016 | Völter, C. J., Sentís, I., & Call, J. (2016). Great apes and children infer causal relations from patterns of variation and covariation. Cognition, 155, 30-43. doi:10.1016/j.cognition.2016.06.009. |
| volter2018intuitive | 2018 | Völter, C. J., & Call, J. (2018). Intuitive optics: What great apes infer from mirrors and shadows. Animal Cognition, 21(4), 493-512. doi:10.1007/s10071-018-1184-0. |
| volter2019chimpanzees | 2019 | Völter, C. J., Mundry, R., Call, J., & Seed, A. M. (2019). Chimpanzees flexibly update working memory contents and show susceptibility to distraction in the self-ordered search task. Proceedings of the Royal Society B: Biological Sciences, 286(1907): 20190715. doi:10.1098/rspb.2019.0715. |
| wolf2019visually | 2019 | Wolf, W., & Tomasello, M. (2019). Visually attending to a video together facilitates great ape social closeness. Proceedings of the Royal Society B: Biological Sciences, 286(1907): 20190488. doi:10.1098/rspb.2019.0488. |
| wolf2020human | 2020 | Wolf, W., & Tomasello, M. (2020). Human children, but not great apes, become socially closer by sharing an experience in common ground. Journal of Experimental Child Psychology, 199: 104930. doi:10.1016/j.jecp.2020.104930. |

*the data contains unpublished data (see details below)

**Unpublished data**

Some trials from brauer2007chimpanzees were not published in the original publication. The trials follow the same methodology as those presented in brauer2008chimpanzees (competitive theory of mind task where a subordinate needs to infer the decision of a competitive partner based on noise cues). See more details in Brauer et al., 2007 and 2008.

**Annotated yaml including all timestamped events and email correspondence entries throughout the standardization process.**

study_id: sanchez2017chimpanzees

scraper_name: Lizbeth Mujica

scraper_comments: ''

year: 2017

reference: 'Sanchez-Amaro, A., Duguid, S., Call, J., & Tomasello, M. (2017). Chimpanzees,

bonobos and children successfully coordinate in conflict situations. Proceedings

of the Royal Society B: Biological Sciences, 284(1856): 20170259. doi:10.1098/rspb.2017.0259.'

abstract: Social animals need to coordinate with others to reap the benefits of groupliving

even when individuals’ interests are misaligned. We compare how chimpanzees, bonobos

and children coordinate their actions with a conspecific in a Snowdrift game, which

provides a model for understanding how organisms coordinate and make decisions under

conflict. In study 1, we presented pairs of chimpanzees, bonobos and children with

an unequal reward distribution. In the critical condition, the preferred reward

could only be obtained by waiting for the partner to act, with the risk that if

no one acted, both would lose the rewards. Apes and children successfully coordinated

to obtain the rewards. Children used a ‘both-partner-pull’ strategy and communicated

during the task, while some apes relied on an ‘only-one-partner-pulls’ strategy

to solve the task, although therewere also signs of strategic behaviour as they

waited for their partner to pull when that strategy led to the preferred reward.

In study 2, we presented pairs of chimpanzees and bonobos with the same set-up as

in study 1 with the addition of a non-social option that provided them with a secure

reward. In this situation, apes had to actively decide between the unequal distribution

and the alternative. In this set-up, apes maximized their rewards by taking their

partners’ potential actions into account. In conclusion, children and apes showed

clear instances of strategic decision-making to maximize their own rewards while

maintaining successful coordination.

keywords: coordination, conflict, Snowdrift, chimpanzees,bonobos, children

any_data_provided: true

current_status: complete - include

internal_reviewer_name: Alex Sanchez

authors:

- author_no: 1

name: Alejandro Sanchez-Amaro

email_current: alex_sanchez@eva.mpg.de

corr_author: true

- author_no: 2

name: Shona Duguid

email_current: null

corr_author: false

- author_no: 3

name: Josep Call

email_current: null

corr_author: false

- author_no: 4

name: Michael Tomasello

email_current: null

corr_author: false

experiments:

- experiment_no: 1

data_provided: partial

reason_no_data: null

reason_no_data_cat: null

grouping: dyads

dropouts_no: null

dropout_names: null

group_id: wkprc_bonobo, wkprc_chimpanzee_a

group_id_excluded: leipzig_children

data_collection_date_provided: true

data_collection_date_source: dataset

data_collection_date: 2014 - 2015

data_collection_date_comments: ''

age_source: calculated

age_source_comments: ' '

- experiment_no: 2

data_provided: true

reason_no_data: null

reason_no_data_cat: null

grouping: dyads

dropouts_no: null

dropout_names: null

group_id: wkprc_bonobo, wkprc_chimpanzee_a

group_id_excluded: null

data_collection_date_provided: true

data_collection_date_source: dataset

data_collection_date: 2015 - 2016

data_collection_date_comments: ''

age_source: calculated

age_source_comments: ' '

domains:

- domain_no: 1

general_domain: competition_and_cooperation

specific_domain: collaboration

cognitive_domain:

social: true

physical: false

events:

- phase: standardization

name: Carin Molenaar

date: 2022-04-01

comment: initial standardization

- phase: internal review

name: Alex Sanchez

date: 2022-11-22

comment: glossary ready to be filled

- phase: standardization

name: Akzira Abuova

date: 2024-01-11

comment: condition/condition_code needs to be renamed; otherwise glossary ready

for author approval

- phase: standardization

name: Carin Molenaar

date: 2024-01-16

comment: redundant columns removed. discrepancy between glossary and glossary_ongoing

columns in experiment 2 resolved

- phase: standardization

name: Carin Molenaar

date: 2024-01-19

comment: added age of participants and added focal to participant

- phase: standardization

name: Akzira Abuova

date: 2024-10-28

comment: the data is complete and can go to the final folder

author_list:

- requested: true

requested_date: 2024-10-28

received: true

received_date: null

emails:

- email_sent_on: null

email_sent_by: null

email_sent_by_email: null

email_sent_to: null

email_sent_to_email: null

email_type: null

email_comments: null

**Annotated yaml only including relevant public metadata information for publication**

**study_id: sanchez2017chimpanzees**

year: 2017

reference: ‘Sanchez-Amaro, A., Duguid, S., Call, J., & Tomasello, M. (2017). Chimpanzees,

bonobos and children successfully coordinate in conflict situations. Proceedings

of the Royal Society B: Biological Sciences, 284(1856): 20170259. Doi:10.1098/rspb.2017.0259.’

abstract: Social animals need to coordinate with others to reap the benefits of groupliving

even when individuals’ interests are misaligned. We compare how chimpanzees, bonobos

and children coordinate their actions with a conspecific in a Snowdrift game, which

provides a model for understanding how organisms coordinate and make decisions under

conflict. In study 1, we presented pairs of chimpanzees, bonobos and children with

an unequal reward distribution. In the critical condition, the preferred reward

could only be obtained by waiting for the partner to act, with the risk that if

no one acted, both would lose the rewards. Apes and children successfully coordinated

to obtain the rewards. Children used a ‘both-partner-pull’ strategy and communicated

during the task, while some apes relied on an ‘only-one-partner-pulls’ strategy

to solve the task, although therewere also signs of strategic behaviour as they

waited for their partner to pull when that strategy led to the preferred reward.

In study 2, we presented pairs of chimpanzees and bonobos with the same set-up as

in study 1 with the addition of a non-social option that provided them with a secure

reward. In this situation, apes had to actively decide between the unequal distribution

and the alternative. In this set-up, apes maximized their rewards by taking their

partners’ potential actions into account. In conclusion, children and apes showed

clear instances of strategic decision-making to maximize their own rewards while

maintaining successful coordination.

Keywords: coordination, conflict, Snowdrift, chimpanzees,bonobos, children

any_data_provided: true

authors:

- author_no: 1

name: Alejandro Sanchez-Amaro

- author_no: 2

name: Shona Duguid

- author_no: 3

name: Josep Call

- author_no: 4

name: Michael Tomasello

experiments:

- experiment_no: 1

data_provided: partial

grouping: dyads

dropouts_no: null

dropout_names: null

group_id: wkprc_bonobo, wkprc_chimpanzee_a

group_id_excluded: eipzig_children

data_collection_date_provided: true

data_collection_date_source: dataset

data_collection_date: 2014 – 2015

data_collection_date_comments: ‘’

age_source: calculated

age_source_comments: ‘ ‘

- experiment_no: 2

data_provided: true

grouping: dyads

dropouts_no: null

dropout_names: null

group_id: wkprc_bonobo, wkprc_chimpanzee_a

group_id_excluded: null

data_collection_date_provided: true

data_collection_date_source: dataset

data_collection_date: 2015 – 2016

data_collection_date_comments: ‘’

age_source: calculated

age_source_comments: ‘ ‘

domains:

- domain_no: 1

general_domain: competition_and_cooperation

specific_domain: collaboration

cognitive_domain:

social: true

physical: false

**Example of data standardization script**

import json

import os

with open('config.json', 'r') as f:

data = json.load(f)

pathway_gen = os.path.abspath(data["python_files"])

pathway_temp = os.path.abspath(data["publications"])

pathway = os.path.join(pathway_temp, "sanchez2017chimpanzees")

original_data_pathway = os.path.join(pathway, "original_data")

complete_path_1 = os.path.join(original_data_pathway, "Sanchez_2017_Dataset_Study1a.csv")

complete_path_2 = os.path.join(original_data_pathway, "Sanchez_2017_Dataset_Study 2.csv")

out_pathway = os.path.join(pathway, "standardized_data")

if not os.path.exists(out_pathway):

os.makedirs(out_pathway)

import pandas as pd

import numpy as np

import pyreadstat

#open original data files

df1 = pd.read_csv(complete_path_1)

df1['experiment_name']="1a"

df2 = pd.read_csv(complete_path_2)

df2['experiment_name']="2"

data_frames=[ df1, df2] ##temporarily combine data frames to facilitate data standardization

for index, x in enumerate(data_frames):

x.columns = map(str.lower, x.columns) ##remove capitalization

x=x.applymap(lambda s: s.lower() if type(s) == str else s) ##remove capitalization

x['study_id']="sanchez2017chimpanzees" ##include study_id variable

x = x.rename(columns={"sex_dyad": "dyad_sex",

"dyad": "dyad_original", ##rename original dyad column to prevent confusion with standardized column

"subject left": "ape", ##initial standardization protocol update subject columns to ape/later updated to participant to include other species when relevant

"subject right": "ape_2"})

x['role']='focal_participant_left' ##clarify roles of participants

x['role_2']='focal_participant_right'

x['ape'] = x['ape'].str.rstrip() ##remove extra spaces after names in both participant columns

x['ape_2'] = x['ape_2'].str.rstrip()

data_frames[index]=x

new_df=data_frames[0]

fulldf = pd.concat(data_frames, ignore_index=True, sort=False)

for x,y in zip(df_name['wrong'],df_name['right']): ##correct common misspellings of participant names

fulldf['ape'].replace(x, y, inplace=True)

fulldf['ape_2'].replace(x, y, inplace=True)

fulldf['dyad']=fulldf.ape.str.cat(fulldf.ape_2, sep='_') ##create standardized dyad column

##add standardized participant information (i.e., sex/species) from WKPRC ape list

comp_path_ape_info = os.path.join(pathway_gen, "apes_includeindatabase.csv")

apedf = pd.read_csv(comp_path_ape_info)

fulldf= fulldf.merge(apedf,left_on='ape', right_on='name', how='left')

comp_path_ape_info_2 = os.path.join(pathway_gen, "apes_includeindatabase_2.csv")

apedf_2 = pd.read_csv(comp_path_ape_info_2)

fulldf= fulldf.merge(apedf_2,left_on='ape_2', right_on='name_2', how='left')

fulldf.columns =fulldf.columns.str.replace(' ', '_') ##replace spaces in variable names with underscore

##update values in variables for clarity by creating temporary columns to allow for easy comparison to ensure updates were made as intended before renaming updated columns for standardized file

code_list=["position_1_reward_in_social_original", "position_5_rewards_in_social_original"]

for index, x in enumerate(code_list):

fulldf[x] = fulldf[x].astype(str)

temp=[]

for entry in fulldf[x]:

if entry == 'in':

entry = "roped_interior_side"

elif entry =='out':

entry = "free_exterior_side"

temp.append(entry)

fulldf = fulldf.assign(temp_col=temp)

fulldf=fulldf.rename(columns={'temp_col': x+'_codes'})

fulldf =fulldf.rename(columns={"position_1_reward_in_social_original_codes":"position_1_reward_in_social",

"position_5_rewards_in_social_original_codes":"position_5_rewards_in_social"})

##update column names to reflect updated standardization protocol that updated subject/ape to participant

fulldf.rename(columns={"subject_left_pull_latency": "participant_left_pull_latency",

"subject_right_pull_latency":"participant_right_pull_latency"}, inplace=True)

##update values in variable columns for clarity

replace_list_1 = ['participant_left_pull_latency','participant_right_pull_latency',

'pull_latency_participant_right', 'pull_latency_participant_left']

for x in replace_list_1:

fulldf[x].replace('np', 'no_pull', inplace=True, regex=True)

##calculate and enter participant ages using date of data collection and participant birth dates as noted by the WKPRC participant list

comp_path_birth_dates = os.path.join(pathway_gen, "apes_age_calculations.csv")

ape_dob = pd.read_csv(comp_path_birth_dates)

fulldf= fulldf.merge(ape_dob,left_on='participant', right_on='name', how='left')

comp_path_birth_dates_2 = os.path.join(pathway_gen, "apes_age_calculations_2.csv")

ape_dob_2 = pd.read_csv(comp_path_birth_dates_2)

fulldf= fulldf.merge(ape_dob_2,left_on='participant_2', right_on='name_2', how='left')

two_participant_lists = [['dodc','dob','age_in_years'],

['dodc_2','dob_2','age_in_years_2']]

for x,y,k in two_participant_lists:

fulldf[x] = fulldf['year'].astype(str) + '-' + fulldf['month'].astype(str) + '-' + fulldf['day'].astype(str)

fulldf[x] = pd.to_datetime(fulldf[x])

fulldf[y] = pd.to_datetime(fulldf[y])

fulldf[k] = (fulldf[x] - fulldf[y]).dt.days//365

##organize data frame in order of standardization protocol in preparation for exporting standardized file

fulldf=fulldf[['study_id', 'experiment_name','year','month','day',

'participant', 'age_in_years','sex', 'role',

'participant_2', 'age_in_years_2','sex_2', 'role_2', 'species', 'dyad', 'dyad_sex',

'phase', 'session', 'trial',

'condition', 'pull',

'focal_participant_left_pull_latency', 'focal_participant_right_pull_latency',

'get_4', 'get_1',

'minimum_latency_to_pull', 'pull_binomial',

'food_in_non_social', 'position_5_rewards_in_social',

'position_1_reward_in_social',

'choice_focal_participant_right','choice_focal_participant_left',

'latency_open_door_right',

'latency_open_door_left',

'pull_latency_focal_participant_right','pull_latency_focal_participant_left', 'get_focal_participant_right', 'get_focal_participant_left' ]]

##split experiments into two files and export both standardized data file and corresponding empty glossary

exp1 = fulldf[fulldf['experiment_name'] == '1a']

exp2 = fulldf[fulldf['experiment_name'] == '2']

experiments = [[exp1, 'sanchez2017chimpanzees_exp1a'],

[ exp2, 'sanchez2017chimpanzees_exp2']]

for x,y in experiments:

x = x.dropna(axis=1, how='all')## drop empty rows/columns

comp_out_path_stand = os.path.join(out_pathway, y+'_standardized.csv')

x.to_csv(comp_out_path_stand, encoding='utf-8-sig', index=False)

##glossaries

names = x.columns.tolist()

df = pd.DataFrame(names)

df = df.rename(columns={0: "column_name"})

df["description"] = ""

studyID_glossary=df[["column_name", "description"]]

comp_out_path_glossary = os.path.join(out_pathway, y+'_glossary.csv')

studyID_glossary.to_csv(comp_out_path_glossary, encoding='utf-8-sig', index=False)

## Additional visualization


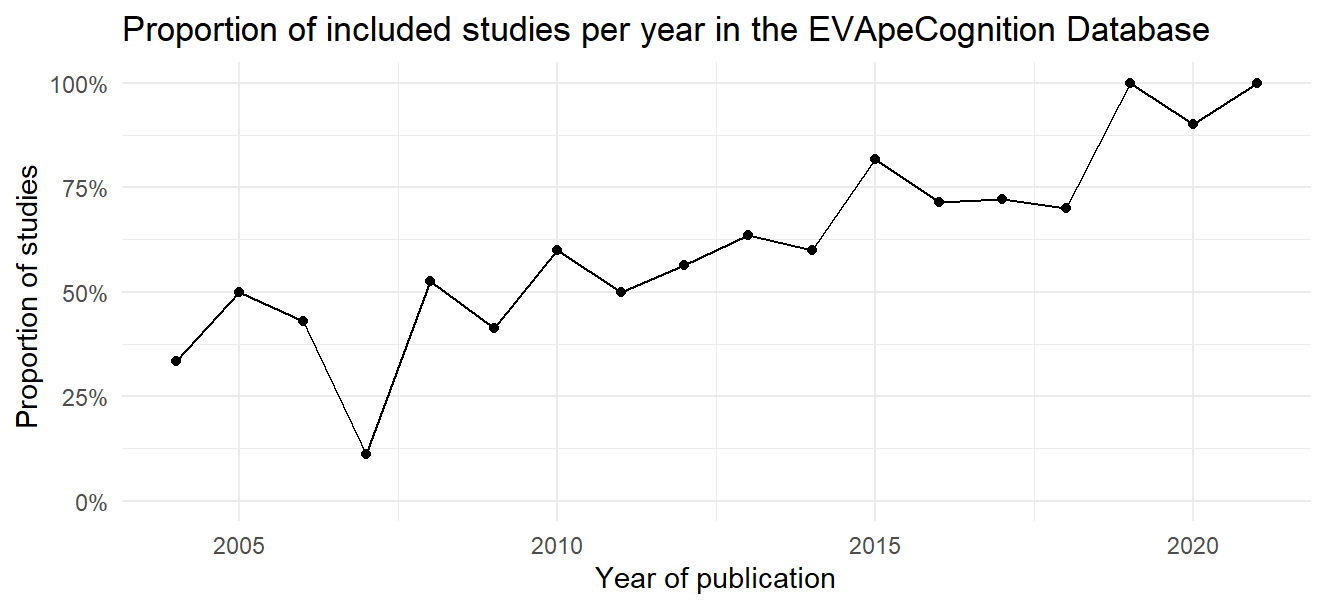


**Figure S1: Proportion of studies recovered for the EVApeCognition Database per year of publication.**
